# Eco-friendly nanocomposite SA/Al_2_O_3_/Ag_2_Mo_2_O_7_ microbeads for fast and sustainable photo-induced adsorption of methylene blue dye from industrial wastewater

**DOI:** 10.1038/s41598-026-61010-x

**Published:** 2026-07-10

**Authors:** Mohamed A. El-Damanhoury, Ahmed H. Mangood, Enas M. Abou-Taleb, Ahmed M. Rashad, Mohamed S. Hellal

**Affiliations:** 1 Chemistry Department, Faculty of Science, Menoufia University, Shebin El Kom, Menoufia Egypt; 2 Water Pollution Research Department, National Research Centre, Cairo, Egypt; 3 Analysis and Evaluation Department, Egyptian Petroleum Research Institute, Nasr City, Cairo, Egypt

**Keywords:** Sodium alginate (SA), Nano-alumina (Al_2_O_3_), Poly silver molybdate (Ag_2_Mo_2_O_7_), Photo-induced adsorption, Microbeads, Methylene blue (MB), Removal, Chemistry, Environmental sciences, Materials science, Nanoscience and technology

## Abstract

The increasing release of hazardous synthetic dye waste poses a serious threat to the environmental and human health. In this study, a novel ternary nanocomposite was designed as an innovative photo-responsive adsorbent by embedding poly silver molybdate (Ag_2_Mo_2_O_7_) and alumina nanoparticles (Al_2_O_3_) into a sodium alginate (SA) polymeric matrix. Designed to efficiently eliminate methylene blue (MB) dye from contaminated water and overcome the separation bottleneck of traditional powdered materials. The SA/Al_2_O_3_/Ag_2_Mo_2_O_7_ microbeads were synthesized via a facile and eco-friendly ionotropic gelation method. Structural and morphological characterization of the prepared nanocomposite was authenticated via X-ray diffraction (XRD) and transmission electron microscopy (TEM), confirmed the successful formation of the ternary nanocomposite with particle size ranging from 10 to 12 nm. The incorporation of Ag_2_Mo_2_O_7_ and Al_2_O_3_ within the alginate polymer matrix significantly enhanced the photo-induced adsorption performance compared to bulk sodium alginate (SA) microbeads. Notably, 1.2 g/L of the microbeads achieved a remarkable 91.78% removal efficiency for methylene blue (MB) dye (77.5 mg/g) within just 40 min under visible light irradiation. In comparison, the nanocomposite demonstrates a significantly higher elimination compared to individual Ag_2_Mo_2_O_7_ and Al_2_O_3_, which exhibited only 60.69% and 56.89%, respectively. Kinetic studies revealed that the removal process obeyed the pseudo-second-order model, while the equilibrium data closely followed the Freundlich isotherm. Thermodynamic parameters confirmed that the photo-induced adsorption process is spontaneous and endothermic. Furthermore, the reusability of the SA/Al_2_O_3_/Ag_2_Mo_2_O_7_ microbeads was tested through five adsorption–desorption cycles, consistently removing more than 73.89% of MB dye. These results demonstrate the practical application of this photo-responsive nanocomposite as an easily recoverable and efficient solution for eliminating toxic cationic dyes from industrial wastewater.

## Introduction

Water is an indispensable resource for human life and for supporting industrial processes. It covers 71% of the Earth’s surface and serves as a universal solvent^1^. However, increasing industrialization has exacerbated water pollution, introducing contaminants such as heavy metals, microplastics, synthetic dyes, and pesticides into aquatic systems^2^. Water pollution now threatens both human health and the environment^3^. Industrial pollution releases toxic compounds into the environment, degrading water quality^4^. Eliminating these toxic contaminants is crucial to provide fresh water that is suitable for drinking and other usages^5,6^. Among these pollutants, dyes such as methylene blue (MB) are frequently encountered contaminants that must be effectively removed to maintain water quality standards. MB is a versatile synthetic dye that has different applications in industry and medicine^7,8^. Conventional wastewater treatment methods include physical processes, chemical treatments, and biological degradation^9^. Physical methods, such as the conventional adsorption technique, offer several distinct advantages, including operational simplicity, high efficiency, and cost-effectiveness. However, these conventional processes often face critical limitations, such as prolonged equilibrium periods for complex organic pollutants, high chemical consumption, excessive operational costs, and the production of toxic sludge^10^. Natural polymers such as sodium alginate (SA) have attracted significant interest in the wastewater treatment field due to their advantageous properties, including high adsorption capacity and compatibility with composite materials^11,12^. Despite their high efficiency, conventional powdered nanomaterials often encounter significant practical challenges. These limitations primarily reside in the difficulty of post-treatment separation and the high risk of secondary environmental pollution due to the nanoparticles leaching. Additionally, the strategic deployment of sodium alginate (SA) nanocomposites as a three-dimensional matrix represents a sophisticated approach to overcoming the kinetic limits and preventing nanoparticles agglomeration^13^. To address the limitations of nanoparticles, the SA/Al_2_O_3_/Ag_2_Mo_2_O_7_ nanocomposite was designed in a stable 3-D microbead form. Furthermore, this macroscopic form facilitates seamless separation and rapid recovery, positioning the composite as a robust, reusable, and scalable candidate for water remediation^14^. A broad spectrum of research has demonstrated the efficiency of alginate biopolymer in water remediation. For example, the calcium alginate beads used to eliminate Basic Black dye which exhibited a maximum adsorption capacity of 57.7 mg/g^15^. Rais Ahmed et al. fabricated the alginate/silver as a nanocomposite for the elimination of Crystal violet dye within 240 min^16^. Zeeshan Ahamad et al. successfully loaded the graphene oxide on Alginate matrix for the removal of Malachite green (MG). The graphene oxide/Alginate hydrogel could remove 90% of the dye in 20 min^17^. Lin Liu et al. studied the adsorption behavior of MB dye on the alginate-halloysite nanotube beads which showed a maximum adsorption capacity of 250 mg/g at temperature of 35 °C^18^. Nano-metal oxides are widely employed as adsorbents due to their efficiency and distinctive physicochemical properties. Among these, aluminum oxide nanoparticles (alumina) stand out as a prominent amphoteric oxide that exists in multiple crystalline states. Alumina is non-toxic, stable, and easy to prepare^19,20^. For decades, alumina has been widely utilized as an adsorbent in the field of environmental applications. Nano-alumina can be effectively used to remove dyes, heavy metals, organic compounds, and antibiotics from industrial wastewater. Notably, nano-alumina has demonstrated high efficiency in adsorbing dyes from textile wastewater^21^. For example, a study reported a maximum phenol adsorption capacity of 16.97 mg/g using synthesized Al_2_O_3_ nanoparticles. Similarly, other research showed that Al_2_O_3_ nanoparticles removed 89.63% of arsenite from aqueous solutions. A study by Hamid Tajizadegan et al. showed that ZnO/Al_2_O_3_ nanocomposite could eliminate 98% of Methyl orange (MO) dye from industrial wastewater in 20 min^22^. Vijaya et al. successfully synthesized Al_2_O_3_ nanoparticles by using a sol–gel procedure, which showed a maximum adsorption capacity for MB of 23.9 mg/g^23^. The Fe_3_O_4_/Al_2_O_3_ hybrid composite was examined as an excellent adsorbent for the removal of Acid black1 (AB1); this composite could remove 90% of AB1 effectively in 12 h^24^. Silver-based photo-responsive materials are distinguished by the hybridization of 4*d* silver and 2*p* orbitals in oxygen. This unique electronic structure results in a narrow bandgap and efficient photogenerated carrier migration, consequently enabling superior photo-induced removal performance across a wide visible light spectrum. Ag-based materials have been regarded as one of the most promising photo-responsive systems, including Ag_2_O, Ag_3_AsO_4_, Ag_3_PO_4_, AgAlO_2_, Ag_2_MoO_4_, and poly silver molybdate (Ag_2_Mo_2_O_7_). Among them, Ag–Mo–O materials are considered highly promising due to their impressive visible light absorption^25^. For example, a high removal efficiency of MB (91.8%) was achieved under visible light irradiation using Ag_2_MoO_4_/Bi_2_MoO_6_ as a photocatalyst, demonstrating its excellent potential for degrading organic contaminants in industrial wastewater^26^. The Au/Ag_2_MoO_4_ nanocomposite was also investigated as a superior photocatalyst for the degradation of MB with up to 99.7% efficiency under UV irradiation in 240 min^27^. The modified form MgS/Ag_2_MoO_4_ nanohybrid was used as photocatalyst for the degradation of MB, which achieved a maximal adsorption efficiency of 90%. Also, the bulk materials of this composite were investigated for the elimination of methylene blue dye. The bulk materials MgS, Ag_2_MoO_4_ can eliminate 41% and 62% of MB from contaminated water, respectively^28^. Poly silver molybdate (Ag_2_Mo_2_O_7_) is widely applied as photo-responsive material to eliminate contaminants from industrial wastewater through a photo-induced removal method. Recently, Ag_2_CrO_4_/Ag_2_Mo_2_O_7_ has been widely investigated as a nano-photocatalyst against 2-naphthol orange (97.8%), Rhodamine B (99.7%), Crystal violet (98.9%), and Methyl orange (56.1%) for about 90 min^29^. Xiaoli Su et al. reported that doping Ag_2_Mo_2_O_7_ with BiOl significantly enhanced the photodegradation of Rhodamine B and tetracycline under visible light irradiation^25^. Shujing Liu et al. attributed the high photocatalytic activity of Tb_2_O_3_/Ag_2_Mo_2_O_7_ composite to its ability to remove MB and tetracycline under visible light^30^. Chun-xue Li observed that the degradation of Rh B by Ag_3_PO_4_/Ag/Ag_2_Mo_2_O_7_ nanowire photocatalyst using visible light^31^. Recently, as documented in the literature cited herein, transition metal-based composites have gained huge attention for environmental remediation purposes. In particular, many previous studies have utilized these materials as efficient photocatalysts to chemically destroy and break down organic dyes in wastewater^4,32,33^. Although those conventional destructive pathways work well, they frequently face practical problems like slow reaction kinetics, the formation of unknown hazardous byproducts, and high energy consumption. To solve these degradation-associated issues, our current work introduces a new design paradigm.

In this work, a ternary nanocomposite was rationally designed by integrating SA, Al_2_O_3_, and Ag_2_Mo_2_O_7_ to create a synergistic effect for wastewater remediation. Sodium alginate (SA) was employed as a flexible, anionic macro-porous 3-D hydrogel matrix to facilitate the sequestration of cationic MB molecules. Meanwhile, Al_2_O_3_ was incorporated as a structural scaffold to provide a high surface area and prevent the agglomeration of the active species. Ag_2_Mo_2_O_7_ serves as the pivotal photo-active component, facilitating rapid charge-carrier separation to prevent charge recombination. Unlike traditional binary composites, this pioneering nanocomposite addresses the critical gap of adsorbent recovery. The central research question of this study is: can the integration of Ag_2_Mo_2_O_7_ and Al_2_O_3_ within an SA matrix transform a traditional adsorbent into a smart photo-responsive adsorbent that overcomes the limitations of conventional adsorbents?

Despite the extensive research on MB removal, this work represents the first-ever synthesis of a ternary nanocomposite in a microbead form. The novelty lies in the strategic integration of these three phases to create a synergistic photo-adsorption mechanism, achieving exceptional removal efficiency with an ultra-fast contact time while ensuring easy mechanical recovery and environmental sustainability.

Based on this rational design, the aim of this research is to develop a sustainable green nanocomposite using sodium alginate (SA) integrated with nano-alumina (Al_2_O_3_) and Ag_2_Mo_2_O_7_ nanoparticles. Herein, the nanocomposite was prepared for the first-time via a facile ionotropic gelation method. The developed nanocomposite was tested for eliminating the cationic dye MB from aquatic solutions using a custom-designed reactor system. Additionally, the nanocomposite can be reused through several adsorption–desorption cycles. The synergistic role of the nanocomposite, the kinetics of photo-induced adsorption, and the mechanism of dye sequestration are discussed in detail, providing a scalable and efficient solution for industrial wastewater treatment.

## Materials and methods

### Materials

In this study, a selection of high-purity materials were utilized to ensure the accuracy and reproducibility of the experimental results. Specifically, sodium alginate (SA), methylene blue (MB), sodium hydroxide (NaOH), and hydrochloric acid (HCl), silver nitrate (AgNO_3_), and aluminum chloride hexahydrate (AlCl_3_·6H_2_O) were all purchased from Sigma-Aldrich. Calcium chloride (CaCl_2_) and sodium molybdate (Na_2_MoO_4_) were procured from Oxford fine chemicals. All materials were used exactly as they received without any further purification. Double-distilled water was employed throughout all experimental stages for solution preparation and glassware cleaning.

### Synthesis of Ag_2_Mo_2_O_7_

In this study, Ag_2_Mo_2_O_7_ nanoparticles were successfully synthesized through a sequence of methodical stages. Initially, two separate acidic solutions were prepared under controlled conditions. The first involved dissolving 0.64 g of Na_2_MoO_4_ in 45 mL of distilled water, utilizing ultrasonication for 10 min to ensure complete dissolution and solution homogeneity. The second solution was prepared by dissolving 0.9 g of AgNO_3_ in 45 mL of distilled water under continuous magnetic stirring for 2 h. Subsequently, the two solutions were slowly combined dropwise under constant stirring for an additional 30 min^34,35^. The resulting precipitate was separated from the solution via filtration, then thoroughly washed with distilled water and ethanol to ensure the removal of any residual ions. Finally, the pale-yellow Ag_2_Mo_2_O_7_ precipitate was dried in an oven at 60 °C for 24 h^30^.

### Synthesis of Al_2_O_3_

Initially, 25 g of aluminum chloride hexahydrate (AlCl_3_·6H_2_O) were dissolved in 500 mL of 0.1 M ethanol solution under magnetic stirring at room temperature. Then, 28% ammonia solution was added dropwise to the aluminum chloride solution until the pH reached 9. The formed gel was continuously stirred for 1 h and allowed to age without stirring for 24 h at room temperature. After the aging step, the gel was dried at 100 °C for 24 h. Finally, the dried product was crushed into a fine powder and calcined at 700 °C for 2 h in a muffle furnace to obtain the crystalline Al_2_O_3_ phase^36^.

### Synthesis of SA/Al_2_O_3_/Ag_2_Mo_2_O_7_ photo-responsive nanocomposite

**Fig. 1:**
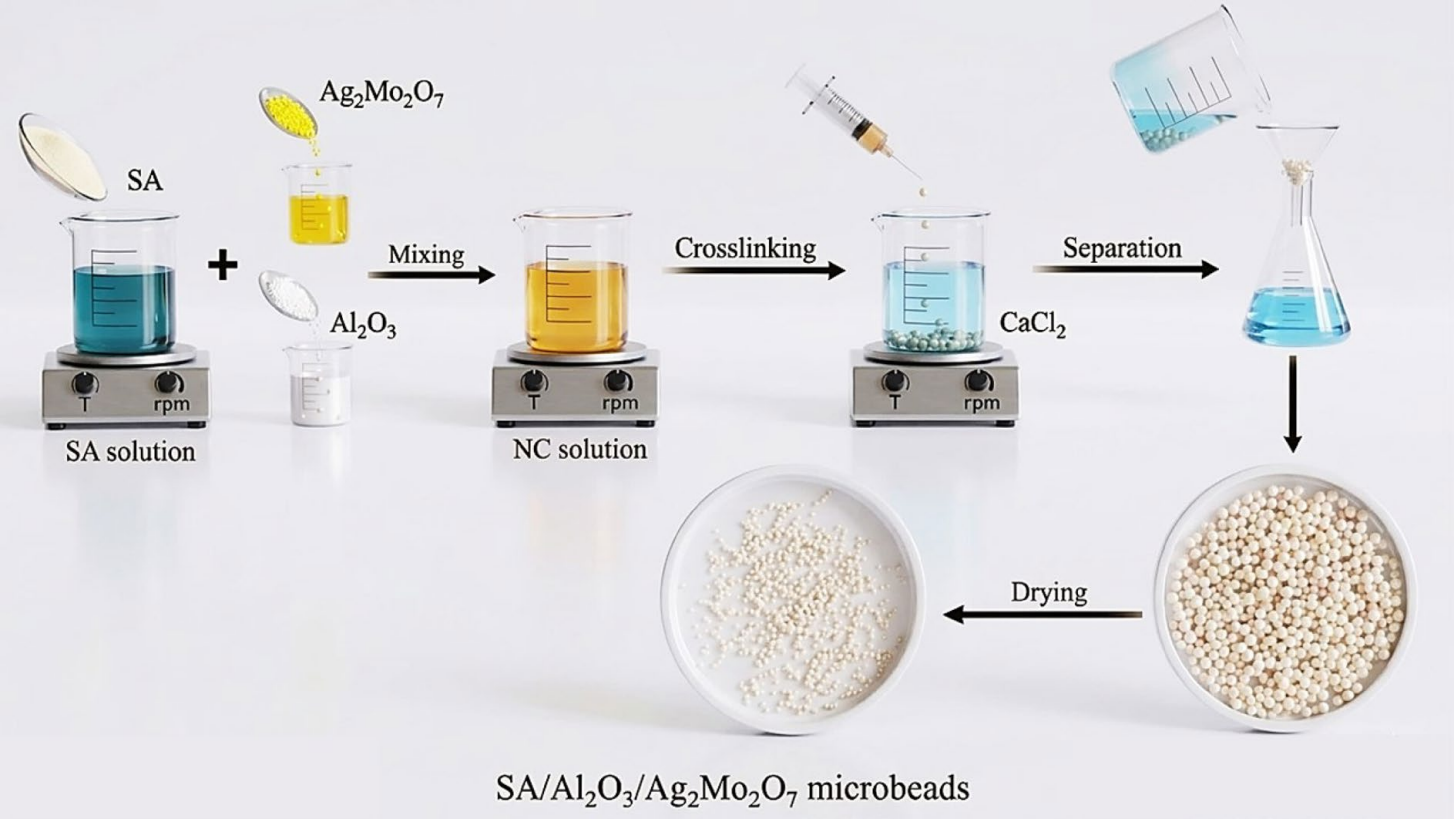
Preparation of SA/Al_2_O_3_/Ag_2_Mo_2_O_7_ microbeads by ionotropic method.

A facile and eco-friendly inotropic gelation method was employed to fabricate the SA/Al_2_O_3_/Ag_2_Mo_2_O_7_ microbeads. The primary advantage of this methodology is its simplicity and the elimination of toxic organic solvents, making it a green, scalable, and cost-effective route for material synthesis. Initially, a uniform solution was crafted by dissolving 0.5 g of sodium alginate (SA) into 25 mL of distilled water, while maintaining a consistent temperature of 45 °C for 1 h. Subsequently, 0.25 g of each of Ag_2_Mo_2_O_7_ and Al_2_O_3_ nanoparticles were dispersed into the SA solution under continuous stirring for 1 h to ensure a homogenous distribution of the active components. The resulting ternary mixture was then transferred into a 3 mL syringe, where the solution was gently dripped into a 200 mL of 2% CaCl_2_ solution to initiate the cross-linking process. The generated microbeads were allowed to immerse in the CaCl_2_ solution for 30 min to ensure complete ionotropic gelation and structural stability. Ultimately, the formed beads were separated, rinsed thoroughly into distilled water to remove any excess ions, and dried in an oven at 60 °C for 24 h^37–40^. The overall synthesis method for these nanocomposite microbeads is illustrated in Fig. 1.

### Instrumental analysis

The chemical functional groups of the prepared samples were identified using a Fourier-transform infrared spectrophotometer (FT-IR, Cary 630, Agilent technologies, USA) within the wavenumber range of 400–4000 cm^−1^. The crystalline structure and phase orientation were analyzed via X-ray diffraction (XRD, X’PERT PRO, PAN analytical, Netherlands). The XRD instrument operated with Ni-filtered C_u_K_α_ radiation (λ = 1.542 Å). The diffraction patterns were collected in the 2θ range from 2° to 80°. The surface morphology and topographic features were examined using a scanning electron microscope (SEM, JSM-5300, JEOL, Japan). To minimize charging effects and enhance conductivity, the samples were mounted on stubs and sputter-coated with a thin layer of gold. The specific surface area and pore volume were determined from nitrogen (N_2_) adsorption–desorption isotherms measured at 77 K using a Micromeritics ASAP 2010 apparatus, applying the Brunauer–Emmett–Teller (BET) method. The internal microstructure and lattice fringes were investigated using high-resolution transmission electron microscopy (HRTEM, JEM-2100, JEOL, Japan) operating at an accelerating voltage of 200 kV. For HRTEM analysis, the sample was prepared by dispersing the material in 30% ethanol solution followed by ultrasonication for 5 min to ensure a homogeneous suspension. Subsequently, one single drop of the mixture was placed on a carbon-coated copper (Cu) grid and allowed to dry completely before imaging.

### Photo-induced adsorption experimental setup and protocol

To distinguish between passive dark adsorption and photo-induced adsorption, a control experiment was initially conducted in the absence of light irradiation by wrapping the reactor in aluminum foil. Subsequently, all further experiments were performed under visible light irradiation.

**Fig. 2:**
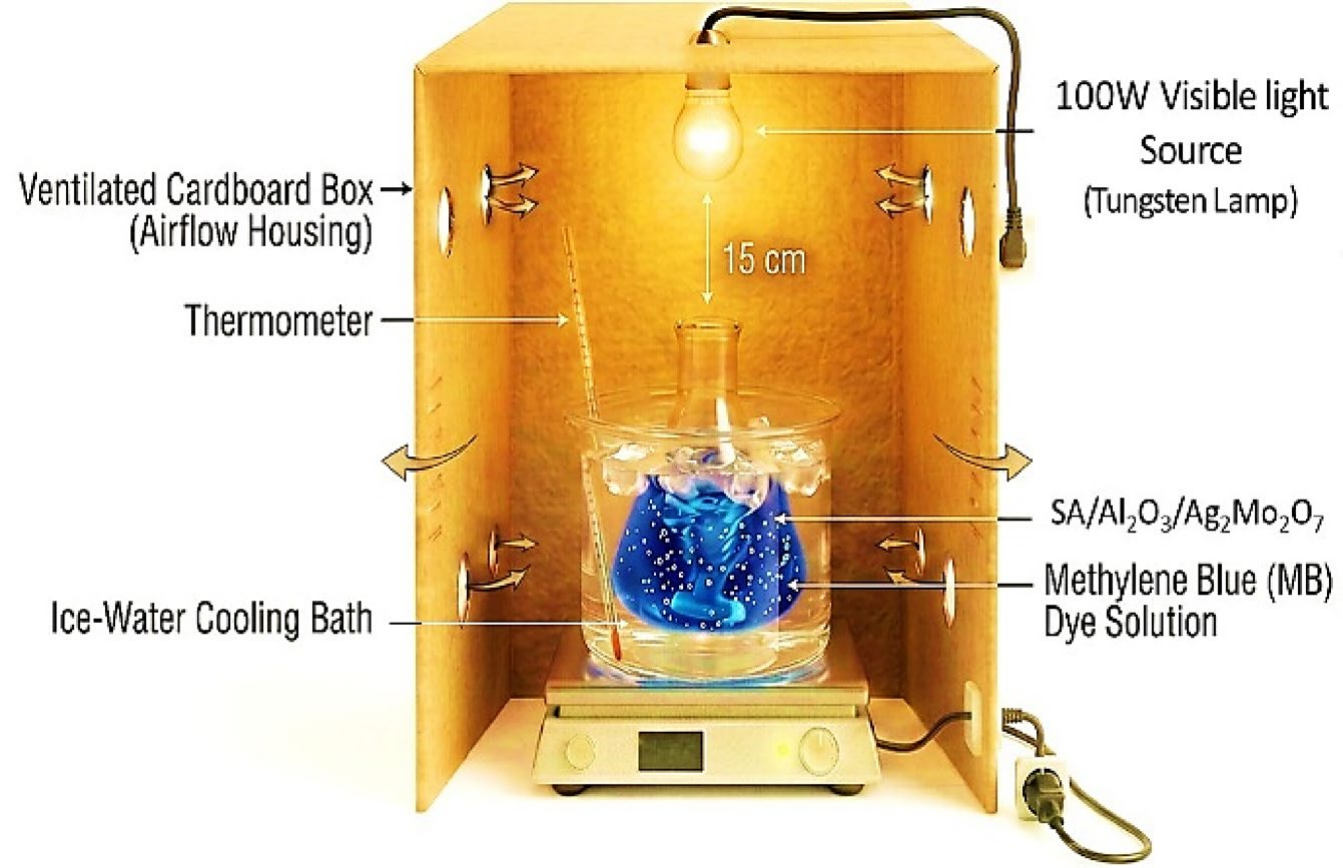
Schematic illustration of the photo-induced adsorption set up for MB removal by using SA/Al_2_O_3_/Ag_2_Mo_2_O_7_ microbeads under visible light.

The photo-induced adsorption experiments were carried out in a custom-designed reactor system to ensure precise control over light intensity, temperature, and mixing homogeneity, as shown in Fig. 2. The reaction was performed in 100 mL cylindrical conical flasks filled with 25 mL of MB solution, which served as the primary reactor. For each experimental run, the dosage of the ternary SA/Al_2_O_3_/Ag_2_Mo_2_O_7_ nanocomposite was varied from 0.4 to 3.6 g/L, with initial MB concentrations ranging between 5 and 85 mg/L. To ensure mixing homogeneity, the reaction mixture was continuously stirred using a magnetic stirrer at a constant speed of 120 rpm. Furthermore, as part of the established operational protocol, the effect of thermal conditions was evaluated by varying the reaction temperature between 25 and 45 °C. For visible light irradiation, a 100 W tungsten filament lamp was positioned at a fixed vertical distance of 15 cm above the solution surface. The calibrated light intensity (irradiance) at the reaction interface was determined to be 2.2 mW/cm^2^.

Thermal stability was strictly maintained to isolate the photochemical response from any thermal effects; the conical flask was immersed in a larger beaker filled with an ice-water cooling bath to dissipate the heat emitted by the light source, as explained in Fig. 2. Furthermore, the irradiation chamber (box) was designed with integrated ventilation openings and kept partially open during the experiments to facilitate natural air convection and prevent heat accumulation. Additionally, experiments were conducted in an air-conditioned laboratory environment to maintain a stable ambient temperature. This dual-cooling strategy ensured that the reaction temperature remained stable at 25 ± 2 °C.

At predetermined time intervals, the flasks were briefly removed. A sample of MB solution (3 mL) was transferred into a 1-cm quartz cell, and the absorbance of the residual MB was measured at λ_max_ = 664 nm using a UV–Vis spectrophotometer. Crucially, due to the macroscopic bead form, the treated solution was easily separated from the adsorbent without the need for energy-intensive centrifugation or filtration. The removal efficiency percentage (R%) was then calculated from Eq. (1). The amount of MB adsorbed at time t and the equilibrium was also determined from Eqs. (2) and (3), respectively^41^.

Overall, this clearly structured operational setup was strictly to ensure the reproducibility and reliability of the photo-induced adsorption performance of the SA/Al_2_O_3_/Ag_2_Mo_2_O_7_ microbeads for methylene blue (MB) removal.

1 $${\text{Removal efficiency }}\left( {\mathrm{R}} \right){\text{ \% }} = \frac{{\left( {C_{o} - C_{t} } \right)}}{{C_{o} }} \times 100$$

2 $${\mathrm{q}}_{{\mathrm{t}}} = \frac{{\left( {C_{o} - C_{t} } \right)}}{m} \times {\mathrm{V}}$$

3 $${\mathrm{q}}_{{\mathrm{e}}} = \frac{{\left( {C_{o} - C_{e} } \right)}}{m} \times {\mathrm{V}}$$

The amounts of MB adsorbed at time t and at equilibrium are represented by q_t_ and q_e_ (mg/g), C_o_ and C_t_ (mg/L) represent the initial and time-dependent concentrations of MB in solution, respectively. C_e_ (mg/L) is the concentration of MB in solution at equilibrium. While, m (g) is the mass of the nanocomposite, and V (L) is the operating volume of the dye solution.

### Reusability study

The ability of the SA/Al_2_O_3_/Ag_2_Mo_2_O_7_ microbeads was tested to be reused over full five cycles of adsorption and desorption. This assessment is crucial for determining the economic viability and sustainability of the developed photo-responsive adsorbent for long-term wastewater treatment. The regenerating the beads that can be reused multiple times, making the treatment more sustainable and cost-effective. The desorption experiments were carried out by using different solutions such as 0.1 M HCl, 0.1 M NaOH, and absolute ethanol under controlled conditions^42^. Among the tested eluents, 0.1 M HCl demonstrated superior desorption performance, making it the preferred choice for regenerating the microbeads^40^. In each experiment, the used beads were immersed in 10 mL of the chosen desorption solution (0.1 M HCl). The mixture was stirred continuously for 6 h to ensure the complete elution of MB molecules from the active sites. Afterwards, the regenerated microbeads were thoroughly rinsed several times with distilled water. Finally, the cleaned beads were carefully dried at 60 °C to be reused in further photo-induced adsorption runs^43^.

## Results and discussion

### Characterization of nanocomposite

#### FT-IR

**Fig. 3:**
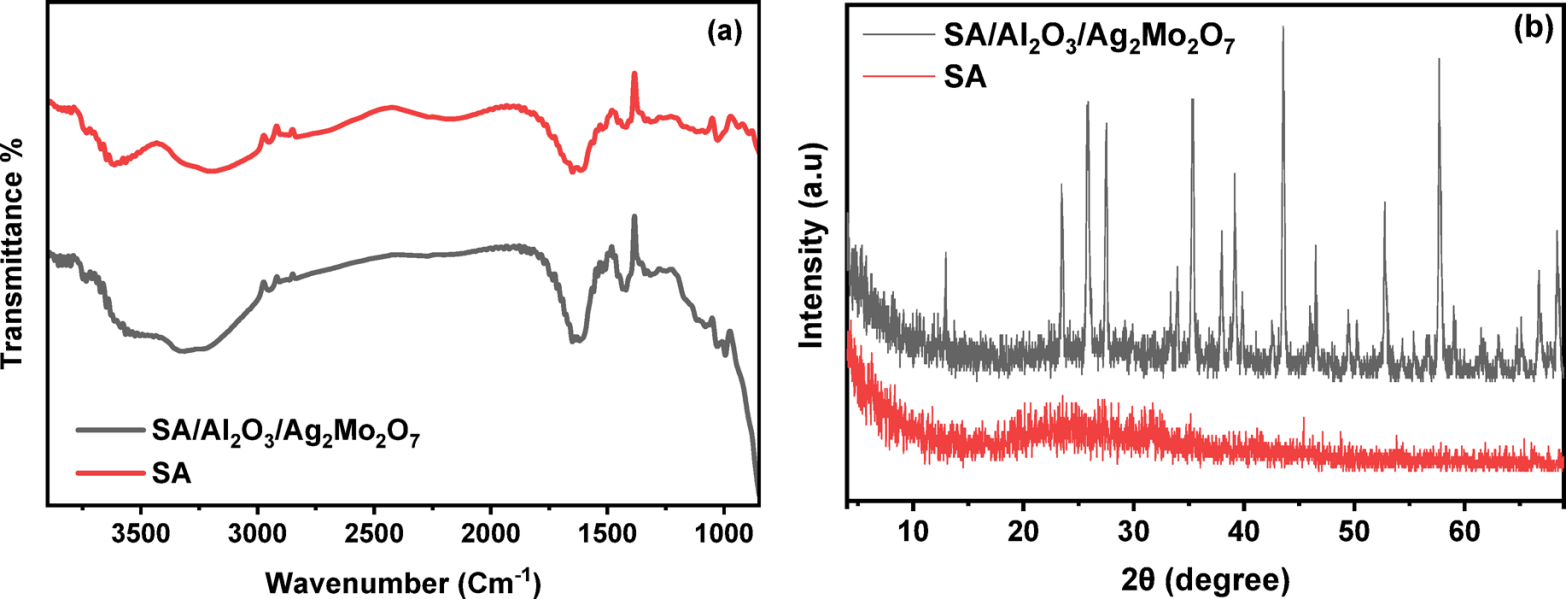
(**a**) FT-IR spectra of SA and SA/Al_2_O_3_/Ag_2_Mo_2_O_7_. (**b**) XRD signals of SA microbeads and SA/Al_2_O_3_/Ag_2_Mo_2_O_7_ nanocomposite microbeads.

FT-IR of the developed nanocomposite provides significant information about its chemical structure and the main functional groups. The FT-IR spectra of the pure sodium alginate (SA) and the synthesized SA/Al_2_O_3_/Ag_2_Mo_2_O_7_ ternary nanocomposite are compared to verify the successful incorporation and interaction of the inorganic phases within the polymeric matrix, as shown in Fig. 3a. The results indicate that the polysaccharide framework of alginate is well preserved within the nanocomposite structure. FT-IR spectra of the nanocomposite show similar functional group assignments as pure sodium alginate. The incorporation of the Al_2_O_3_ and Ag_2_Mo_2_O_7_ phases in the sodium alginate matrix induces significant spectral modifications, confirming the formation of this hybrid nanocomposite.

The primary characteristic of sodium alginate is broad and intense absorption band at approximately 3612 cm^−1^, which is assigned to the hydrogen-bonded hydroxyl groups (–OH). In the ternary nanocomposite, this band exhibits a significant broadening and noticeable shift to lower wavenumber (3313 cm^−1^). This bathochromic shift is a clear indicator of the involvement of hydroxyl groups in strong coordination of hydrogen bonding with the Al_2_O_3_ and Ag_2_Mo_2_O_7_ nanoparticles^13,44^. Furthermore, the asymmetric stretching of carboxylate groups (–COO^−^) appears in alginate and the nanocomposite at 1649 cm^−1^. These carboxylate peaks are characteristic of alginate. The change in intensity of this peak, reflecting interactions or crosslinking with other materials. This suggests a strong electrostatic interaction and chemical anchoring between the carboxylate groups of the alginate backbone and the metallic sites of Al and Ag. This interaction facilitates the stabilization of the inorganic phases within the polymer matrix^45^. Moreover, the structural integrity of the polysaccharide backbone is confirmed by the aliphatic C–H stretching vibration peaks appearing between 2913 and 2964 cm^−1^, alongside the characteristic glycosidic C–O–C and ether C–O linkages identified in 1020–1033 cm^−1^ region. The successful integration of the inorganic components is further evidenced by the emergence of new specific vibrational signatures in the fingerprint region. A sharp and distinct peak at 994 cm^−1^ is assigned to the terminal Mo=O stretching vibrations in the di molybdate (Mo_2_O_7_^−2^) clusters^27,46^. Additionally, a new spectral peak around 800 cm^−1^ is attributed to the formation of Ag–O bonds, confirming the successful capping and interfacial interaction of the silver nanoparticles with the alginate chains^47^. The spectrum of the ternary nanocomposite reveals enhanced absorption and broadening in the 750–850 cm^−1^ range. This spectral feature is attributed to the overlapping of Al–O stretching modes from Al_2_O_3_ lattice and Mo–O–Mo vibrations inherent to Ag_2_Mo_2_O_7_ phase. The presence of these combined peaks confirms the successful heterostructure synthesis of the nanocomposite.

The peaks observed in the developed nanocomposite spectrum showed a synergistic combination of the components, demonstrating strong interactions between the sodium alginate (SA) biopolymer and the inorganic constituents of Al_2_O_3_ and Ag_2_Mo_2_O_7_ nanoparticles. In summary, while XRD confirms the phase purity and crystalline retention of the inorganic compounds, the FT-IR results provide the molecular evidence for their chemical anchoring to the polymeric backbone. Together, these techniques verify the successful formation of a robust, integrated ternary hybrid system.

#### XRD

The phase purity and structural crystallinity of the sodium alginate (SA) and the synthesized ternary nanocomposite were meticulously interrogated using X-ray diffraction (XRD), with the resulting diffractogram presented in Fig. 3b. The diffractogram of the pure sodium alginate (SA) exhibits a characteristic broad, non-crystalline, and amorphous halo in the low-angle region about 2θ = 15°–30°. This broad amorphous signature is the definitive fingerprint of the sodium alginate (SA) matrix. In the ternary nanocomposite, the XRD pattern reveals a hybrid structure characterized by the coexistence of this amorphous polymeric background and distinct crystalline reflections, as observed in Fig. 3b. This confirms that the sodium alginate serves as the polymeric backbone for the inorganic components. The primary set of high-intensity, dominant peaks observed at 2θ approx. 25°, 35°, 37°, 43°, 46°, 57°, 61°, and 67°. These peaks exhibit a perfect match with the standard diffraction pattern of α-Al_2_O_3_ (reference code of 04-008-3293). Furthermore, a secondary set of discrete, lower-intensity peaks is clearly resolved in the 2θ range of 25°–35°, corresponding to the poly silver molybdate (Ag_2_Mo_2_O_7_) phase with reference code of JCPDS 00-021-1339.

While XRD primarily confirms the structural integrity and phase purity of the inorganic constituents, the noticeable preservation of the SA amorphous halo alongside the sharp crystalline peaks suggests that the nanoparticles are effectively hosted and stabilized within the polymeric scaffold. The lack of significant peak shifts or additional impurity phases indicates that the SA matrix serves as a protective medium that prevents the agglomeration of the embedded Al_2_O_3_ and Ag_2_Mo_2_O_7_ nanoparticles, maintaining their phototactically active surfaces. This structural synergy, further supported by the chemical interactions identified in the FT-IR analysis, confirms the successful fabrication of a stable hybrid ternary nanocomposite.

#### SEM

**Fig. 4:**
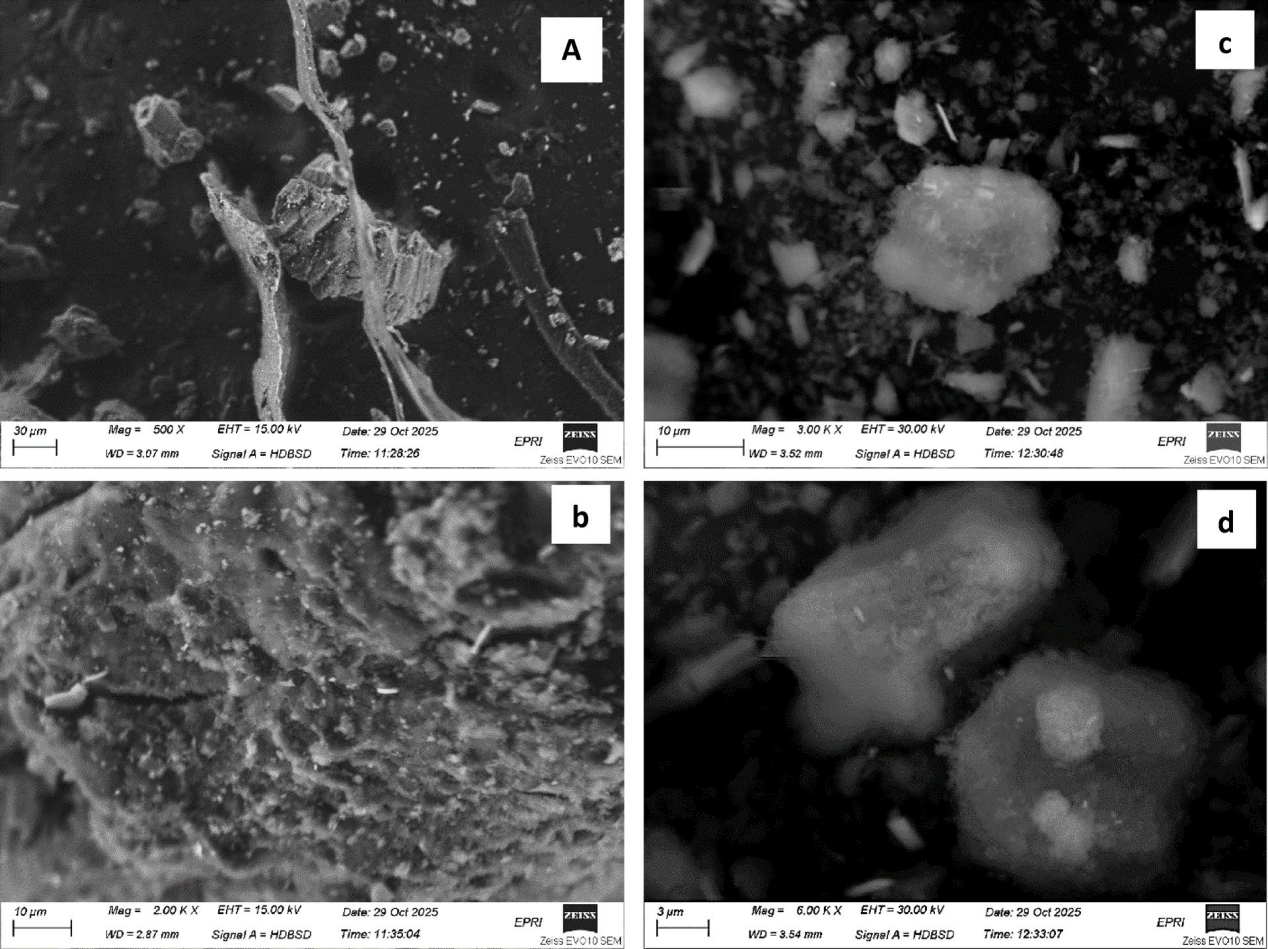
SEM images (**a**,**b**) SA and (**c**,**d**) SA/Al_2_O_3_/Ag_2_Mo_2_O_7_ nanocomposite microbeads.

Based on SEM analysis, the comparative images distinctly delineate the morphological differences in the surface structure of pure sodium alginate (SA) beads versus the synthesized SA/Al_2_O_3_/Ag_2_Mo_2_O_7_ nanocomposite beads. The SA beads display a relatively homogeneous and spherical morphology with a porous or textured surface, as seen in Fig. 4a and b. In contrast, the nanocomposite demonstrates a complex and heterogeneous structure, indicating the effective dispersion of metal oxides into the polymer matrix. This incorporation increases the active sites into the nanocomposite surface, improving its removal capacity for pollutant remediation.

#### EDX

**Fig. 5:**
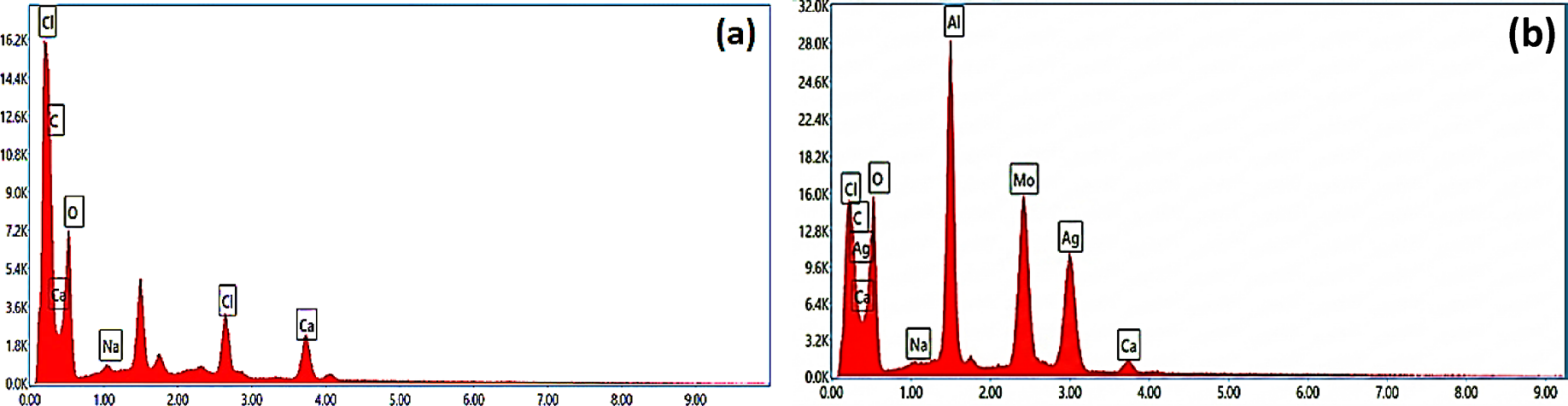
EDX images (**a**) SA and (**b**) SA/Al_2_O_3_/Ag_2_Mo_2_O_7_ nanocomposite.

Elemental analysis of sodium alginate (SA) biopolymer and the SA/Al_2_O_3_/Ag_2_Mo_2_O_7_ nanocomposite showed differences in the constituents of these components obtained using the Energy-Dispersive X-ray (EDX) spectroscopy (Fig. 5). Elemental analysis of the sodium alginate microbeads showed the presence of carbon elements (45.18%), oxygen (43.65%), sodium (2.28%), calcium (4.76%), and chlorine (4.13%)^48^. The EDX spectrum of the ternary nanocomposite confirms the coexistence of all constituent elements, providing direct evidence for the formation of the SA/Al_2_O_3_/Ag_2_Mo_2_O_7_ hybrid nanocomposite. The quantitative analysis revealed the following weight percentages (wt. %) of SA/Al_2_O_3_/Ag_2_Mo_2_O_7_ nanocomposite, containing elements of Mo (19.42%), calcium (1.25%), Al (26.3%), Ag (14.92%), sodium (1.31%), oxygen (21.3%), and carbon (15.5%). The high weight percentages of Al, Ag, and Mo relative to the polymer matrix indicate the successful and dense immobilization of the Al_2_O_3_ and Ag_2_Mo_2_O_7_ nanoparticles. This elemental profile is in excellent agreement with the XRD and FT-IR findings.

#### TEM

**Fig. 6:**
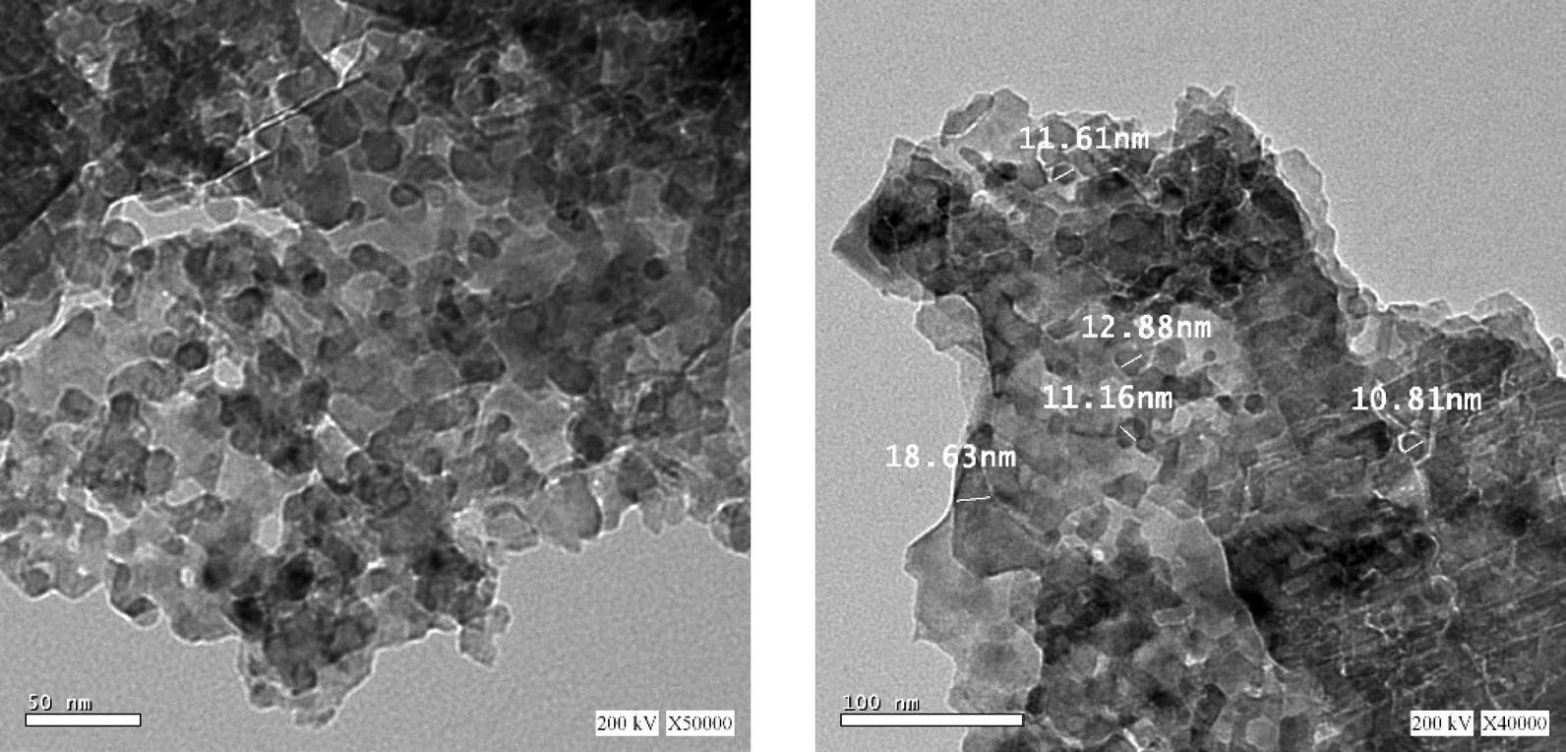
TEM images of SA/Al_2_O_3_/Ag_2_Mo_2_O_7_ nanocomposite microbeads.

The high-resolution transmission electron microscopy (TEM) was utilized to provide a definitive visualization of the SA/Al_2_O_3_/Ag_2_Mo_2_O_7_ ternary nanocomposite structure. The TEM images clearly display the complex nanoscale structure of the nanocomposite. As observed in Fig. 6, the nanocomposite consists of nanosized particles with particle size with a range of diameters between approximately 10 and 12 nm. The micrographs reveal a sophisticated hybrid assembly characterized by the coexistence of diverse morphologies, specifically potato-like spherical, core–shell, nanostructure and an intricate network of elongated nanowires. These nanowires can form networks within nanocomposite matrices, which are important for understanding the material’s dielectric and electrochemical properties^49^.

#### BET

**Fig. 7:**
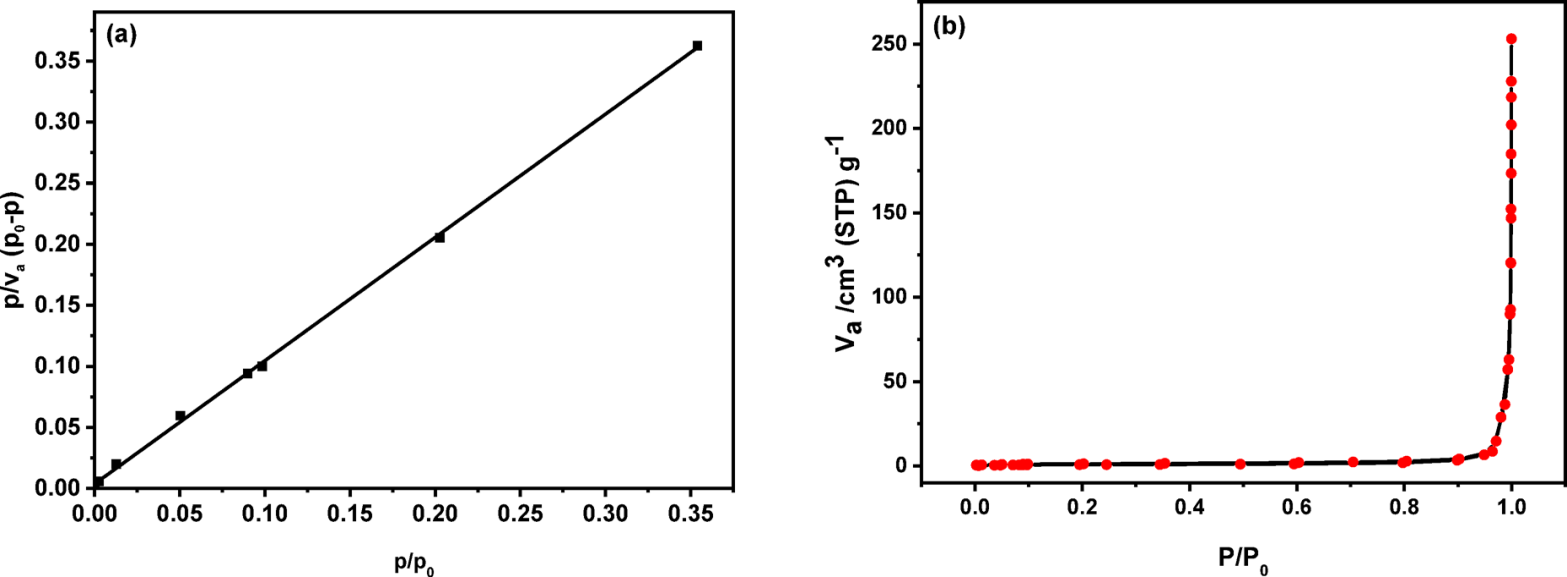
(**a**) linear BET plot (**b**) N_2_ adsorption–desorption isotherm of the SA/Al_2_O_3_/Ag_2_Mo_2_O_7_ nanocomposite.

The textural characteristics of the SA/Al_2_O_3_/Ag_2_Mo_2_O_7_ ternary nanocomposite, including its specific surface area (S_BET_), total pore volume (V_p_), and pore size distribution, were rigorously evaluated using N_2_ adsorption–desorption isotherms. The specific surface area is a critical parameter that directly reflects the density of available active sites for photo-adsorption reactions. As illustrated in Fig. 7, the synthesized nanocomposite possesses a specific surface area of 4.32 m^2^/g and a total pore volume of 0.069 cm^3^/g. While these values reflect a relatively low surface area, a phenomenon frequently observed in molybdenum oxide-based materials. This reduction is primarily attributed to the dense nature of the dried alginate nanocomposites and the large size of the microbeads compared to fine powders. The nitrogen adsorption–desorption profile follows a type II isotherm, which is a definitive indicator of a macro-porous structure according to IUPAC classification. The average pore diameter was determined to be 64.06 nm. This confirms a macroporous framework, which is highly advantageous for wastewater treatment applications. Such large pores facilitate the efficient intra-particle diffusion of large molecules, such as MB within the composite matrix.

### Comparative assessment of MB Photolysis, dark adsorption, and photo-induced adsorption pathways

To understand the synergistic effect of the fabricated ternary nanocomposite and the distinct role of visible light irradiation on MB removal, control experiments were performed under standardized conditions (contact time = 40 min, [MB]_o_ = 13.86 mg/L, V = 25 mL, T = 30 °C, and visible light irradiation using a tungsten lamp). Initially, the direct photolysis of the pure MB dye solution under visible light without any nanocomposite microbeads was investigated at both pH 8 and 12. At pH 8, the direct photolysis of MB dye was completely negligible, exhibiting a removal efficiency of only 3.7%, confirming that light irradiation alone cannot destroy or decolorize the dye molecules. This is in close agreement with previous literature, which documents that direct photolysis of MB under light exposure is completely negligible in acidic and neutral media (around pH 8), confirming the high photostability of the dye molecules under standard conditions^50^. However, at an extreme alkaline medium (such as pH 12), spontaneous photolysis can take place due to proton-producing reactions and chromophore degradation. The direct photolysis leads to self-decolorization of the dye by a slight increase in removal efficiency of 9% under visible light at pH 12. This finding confirms that light irradiation alone cannot induce dye color removal.

To conclusively demonstrates the synergistic impact, parallel control experiments were conducted using 1.2 g/L of the developed nanocomposite at the same conditions. At pH 8, the removal efficiency was 91.78% by using our nanocomposite under visible light. Conversely, at pH 12, the overall removal achieved 99.94% efficiency by using our nanocomposite under visible light irradiation. Consequently, the synergistic combination of visible light and our ternary nanocomposite is exclusively driven by the ultra-fast photo-induced adsorption of the SA/Al_2_O_3_/Ag_2_Mo_2_O_7_ microbeads. Therefore, a mild and safe environment of pH 8 was strictly selected for all practical investigations, completely avoiding any alkaline-induced photolysis interference.

**Fig. 8:**
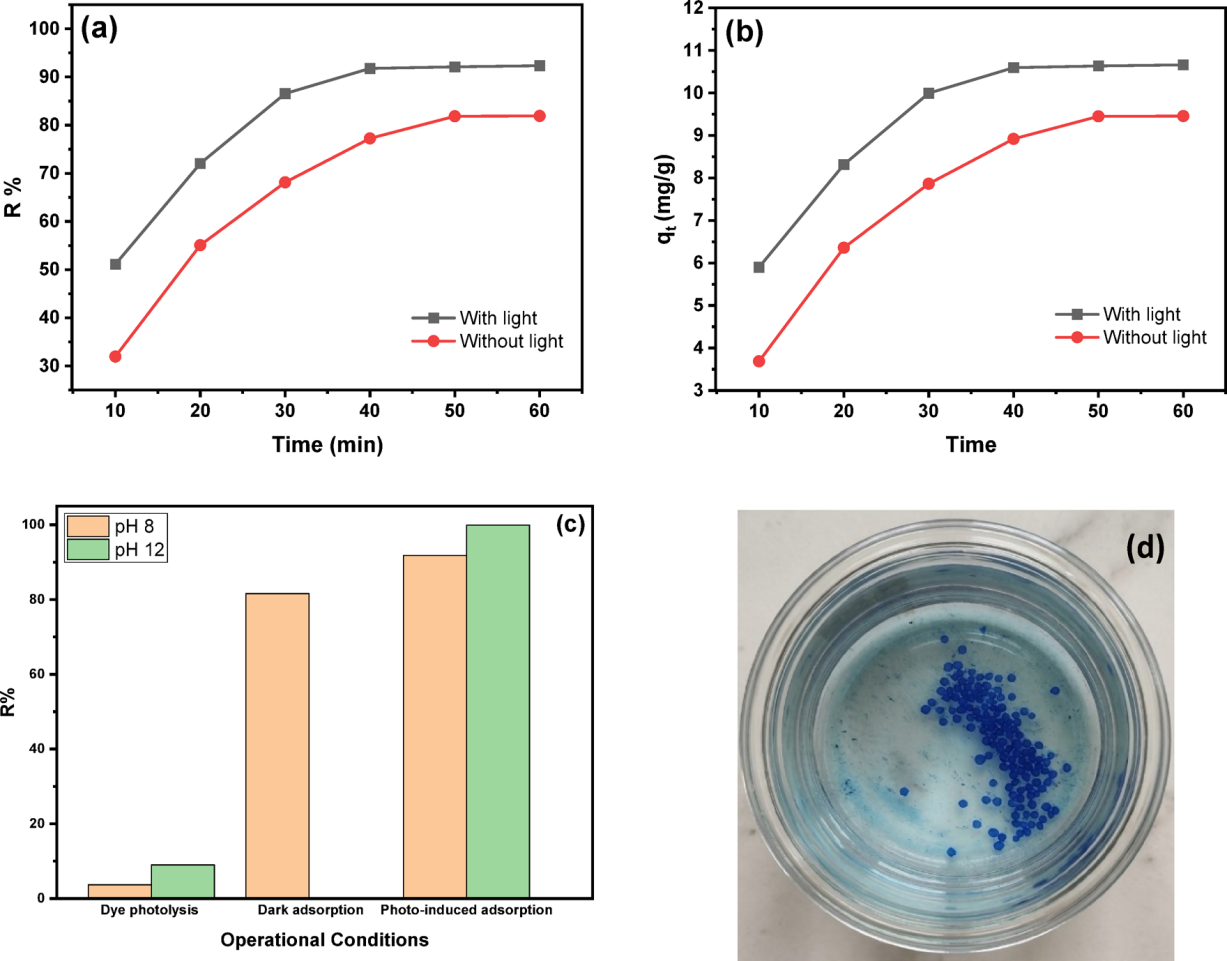
(**a**,**b**) Comparative kinetic profiles and removal efficency under visible light versus without light irradiation. [MB]_o_ = 13.86 mg/L, dose = 1.2 g/L at 30 °C. (**c**) Comparative chart of MB removal efficiency (R%) under different opertational conditions. (**d**) Photogrphic evidence of the retrieved SA/Al_2_O_3_/Ag_2_Mo_2_O_7_ microbeads after photo-induced adsorption.

To further differentiate between conventional dark adsorption and photo-induced adsorption, control experiments were carried out with and without light irradiation using the SA/Al_2_O_3_/Ag_2_Mo_2_O_7_ nanocomposite beads under fixed conditions include adsorbent dosage = 1.2 g/L, contact time = 40 min, [MB]_o_ = 13.86 mg/L, pH = 8, and T = 30 °C. As illustrated in Fig. 8a and b, a remarkable disparity in performance was recorded between the dark and light-driven pathways. Without light irradiation (dark adsorption), the nanocomposite exhibited a standard adsorption behavior with relatively lower removal efficiency and limited adsorption capacity (q_t_), reaching an equilibrium state at approximately 81.6%. Conversely, upon exposure to visible light (photo-induced adsorption experiment), the removal efficiency and q_t_ values were dramatically enhanced, achieving a significant uptake of 91.78% within the same removal period.

As shown in the photographic inserts in Fig. 8d, the microbeads transformed into a deep blue color after the experiment conducted with light irradiation. This visual change serves as tangible proof of a photo-induced adsorption mechanism. The color retention on the microbeads confirms that the methylene blue molecules are physically or chemically sequestered and retained intact on the photo-adsorbent surface, rather than being mineralized into colorless by-products.

As complied in Table 1, comparing the three control systems highlights the clear synergistic performance of the developed nanocomposite. The dye solution alone without any nanocomposite microbeads exhibited negligible photolysis under visible light, confirming its structural stability. Furthermore, while the conventional dark adsorption reached a removal of 81.6%, the photo-induced adsorption pathway demonstrated a high removal efficiency of 91.78%. This significant enhancement mathematically demonstrates that visible light acts as an effective surface activator, successfully expanding the interfacial adsorptive capacity of the ternary nanocomposite. Although the transition from dark adsorption to photo-induced adsorption shows a moderate increase (from 81.6 to 91.78%), this enhancement is highly significant from environmental and economic perspectives. Operating under visible light utilizes an abundant, naturally available, and cost-free energy source, thereby achieving a superior removal efficiency (> 91%) without any secondary energy consumption. Consequently, this enhancement successfully transitions the developed system into a highly-efficient, green, and sustainably driven remediation process, making it practically feasible for large-scale applications.

**Table 1:** Control experiments for MB removal optimization under different systems.

Experiment	System components	Light condition	Removal%
Dye photolysis	MB solution alone (Blank)	Visible light	3.7% at pH 8 9% at pH 12
Dark adsorption	MB solution + nanocomposite	Without light (total darkness)	81.6%,
Photo-induced adsorption	MB solution + nanocomposite	Visible light	91.78%

### Impacts of several parameters on MB removal

#### Selection of the optimal prepared nanocomposite composition

**Fig. 9:**
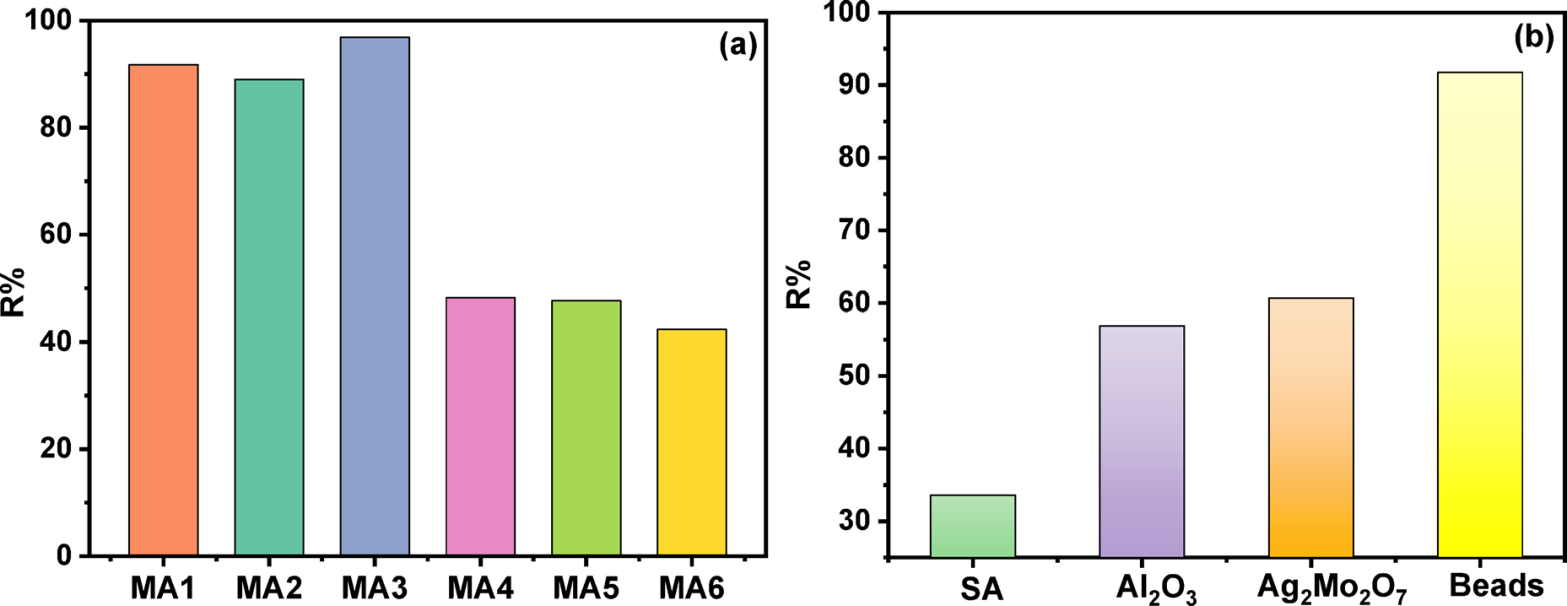
(**a**) Effect of ratio content on the removal of MB. [MB]_o_ = 13.86 mg/L, dose = 1.2 g/L at 30 °C.

The mass ratio of each component plays a crucial role in determining the efficiency of the nanocomposite on the dye removal. Varying the proportion of the components SA, Al_2_O_3_, and Ag_2_Mo_2_O_7_ in the nanocomposite formulation during its preparation under controlled experimental conditions. The prepared nanocomposite microbeads were coded as MA, representing modified alginate matrices incorporated with different loading of Al_2_O_3_ and Ag_2_Mo_2_O_7_. Table 2 summarizes the mass ratio used for each composition, showing the percentage contribution of SA, Al_2_O_3_, and Ag_2_Mo_2_O_7_ in the composite. The impact of the developed nanocomposite mass ratio on the photo-induced adsorptive removal of MB was systematically evaluated under fixed removal conditions. Different compositions were tested to determine the optimal ratio, as shown in Fig. 9. The results revealed that increasing the poly silver molybdate content significantly enhanced MB removal. It was observed that the nanocomposite (MA3) containing the highest mass ratio of Ag_2_Mo_2_O_7_ exhibited the maximum removal efficiency (96.62%). This optimal performance was achieved under the following conditions: an adsorbent dosage of 1.2 g/L, initial MB concentrations of 13.86 mg/L, pH 7.6, and time of 40 min, while maintaining the temperature at 30 °C. In contract, the MA5 and MA6 showed considerably lower removal (47.6% and 42.3%, respectively). Based on these findings, the developed nanocomposite (MA3) was selected for further optimization studies under controlled experimental parameters.

**Table 2:** Several composition of the synthezied nanocomposite with varying component ratios.

Code	SA content (wt%)	Al_2_O_3_ NPs content (wt%)	Ag_2_Mo_2_O_7_ NPs content (wt%)
MA1	20%	60%	20%
MA2	20%	40%	40%
MA3	20%	20%	60%
MA4	40%	40%	20%
MA5	40%	20%	40%
MA6	60%	20%	20%

In addition, the removal percentages of MB using SA microbeads, pure Al_2_O_3_, pure Ag_2_Mo_2_O_7_, and the nanocomposite microbeads were 33.58%, 56.89%, 60.69%, and 91.78%, respectively. Based on these results, the nanocomposite (MA3) was chosen the best prepared adsorbent for further investigation to study different factors effect on the removal efficiency of MB.

#### Impact of contact time

**Fig. 10:**
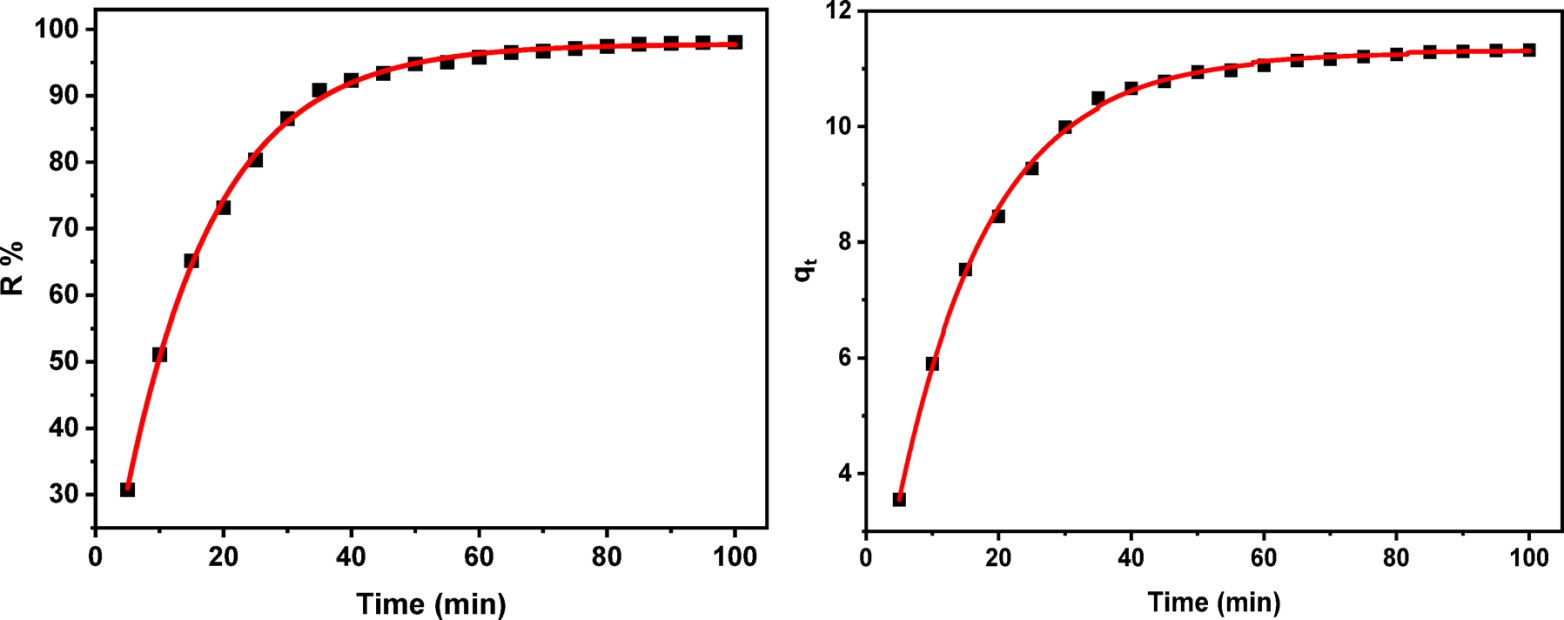
Effect of contact time on (**a**) the removal effeiciency of MB. (**b**) Adsorption capacity of MB with several time intervals. [MB]_o_ = 13.86 mg/L, dose = 1.2 g/L at 30 °C.

The most influential factor controlling removal efficiency is the contact time between the adsorbate and the adsorbent. In this study, the SA/Al_2_O_3_/Ag_2_Mo_2_O_7_ nanocomposite microbeads were examined for MB removal over various time intervals. Figure 10 illustrates how the removal capacity of MB on the developed microbeads changes with incubation time. The experiments were carried out at 30 °C, [MB]_o_ = 13.86 mg/L, and pH = 8.

The process exhibited a three-stage characteristic, starting with an initial rapid phase followed by a slower approach to equilibrium. Initially, the removal was rapid during the first 15 min, representing the bulk diffusion stage, representing the bulk diffusion stage. In this initial stage, the dye molecules migrate rapidly from the aqueous solution to the external surface of the microbeads due to the abundance of available active sites on the nanocomposite surface. As the process continued, the removal rate entered the second stage, a more gradual stage characterized by intra-particle diffusion. During this phase, the MB molecules began to penetrate through the external boundary layer and diffuse into the internal porous network of the microbeads’ structure. This stage is notably slower than the first due to the increased resistance to mass transfer within the microbeads’ matrix. The synergistic effect between the photo-active Ag_2_Mo_2_O_7_ phase and the incident visible light facilitates the continuous sequestration of the dye within these internal active sites. Finally, the removal process reached the steady-state equilibrium phase at approximately 40 min, marking the third and final stage of the process. Beyond this duration, the removal efficiency exhibited a plateau, as the active sites reached a state of saturation. This slowdown may be due to the thinning of the diffusion layer and the saturation of binding sites. Increasing the contact time allows more pollutant molecules to interact with the nanocomposite surface, leading to the highest removal efficiency^51,52^. Therefore, the optimum time of MB removal by the developed microbeads is 40 min. To investigate the mechanism of photo-induced adsorption, the experimental data were examined by using various kinetic models.

#### Impact of microbeads Dosage

**Fig. 11:**
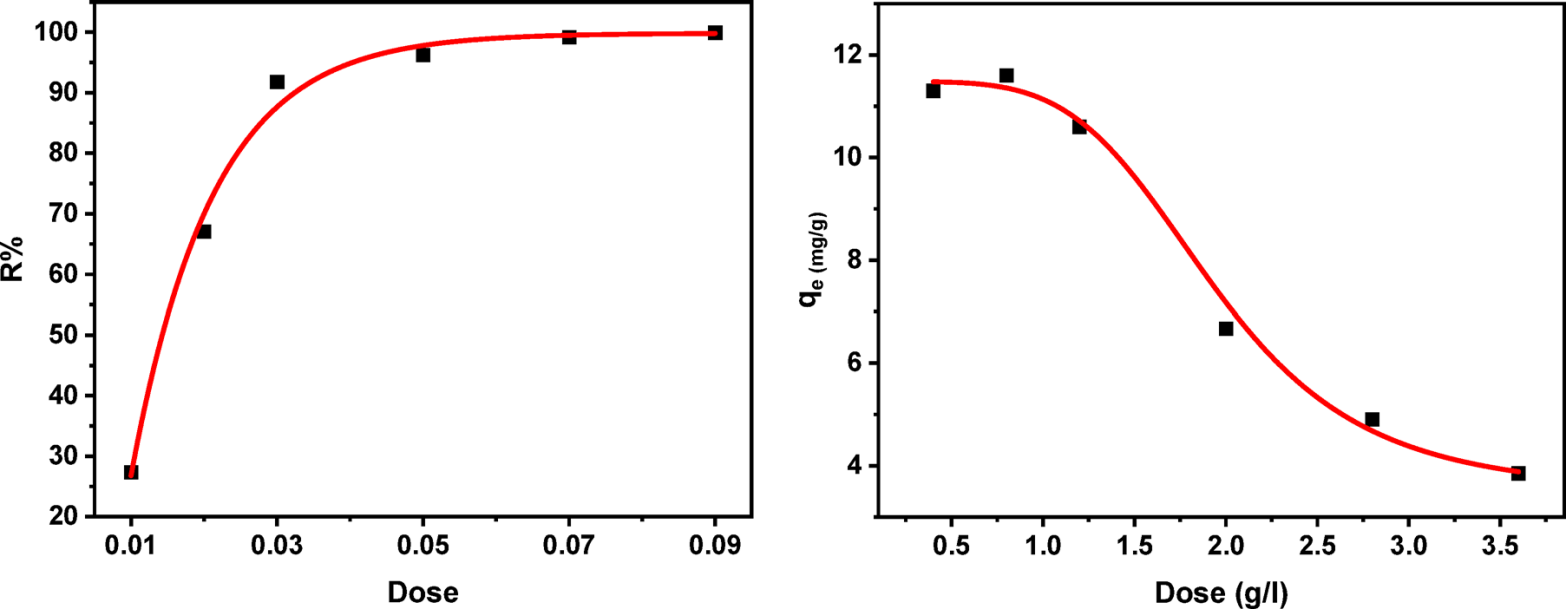
Effect of dose on the removal of MB*.* [MB]_o_ = 13.86 mg/L at 30 °C.

The used amount of the nanocomposite is directly influencing the operational cost of the process. To assess this impact, different quantities of the developed SA/Al_2_O_3_/Ag_2_Mo_2_O_7_ microbeads (from 0.4 to 3.6 g/L) were tested under fixed conditions including Contact time = 40 min, [MB]_o_ = 13.86 mg/L, pH = 8, and T = 30 °C. As shown in Fig. 11, increasing the nanocomposite dose from 0.4 to 3.6 g/L enhanced MB removal from 27.33 to 99.94%. Increasing the amount of nanocomposite raises the number of active sites available on its surface for MB molecules^9^. The maximum removal was achieved at a dose of 3.6 g/L due to the large number of available active sites. Based on these findings, a dose of 1.2 g/L (which achieved 91.78% removal) was chosen as the optimal dosage to study the effect of different factors on MB removal, as it provides a balance between high efficiency and economic feasibility^53,54^.

#### Impact of initial MB concentration

**Fig. 12:**
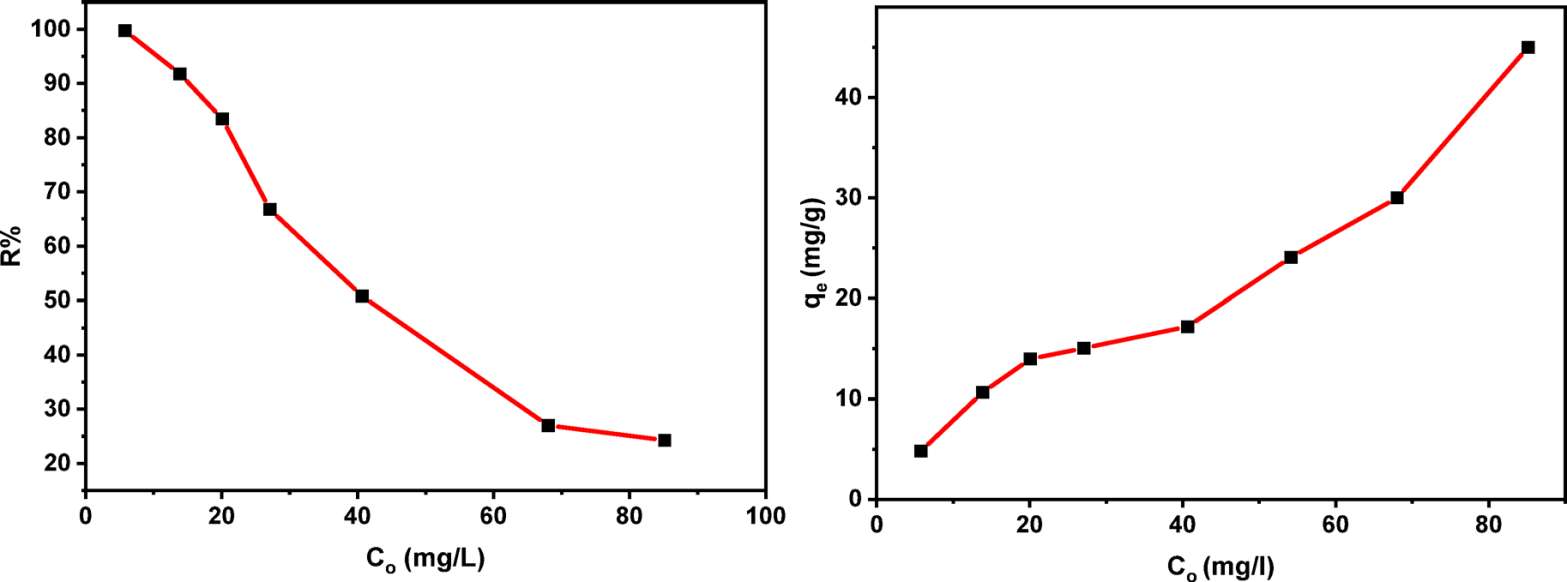
Effect of initial MB concentration on the removal efficiency (%) and equilibrium adsorption capacity (q_e_). (Conditions: dosage 1.2 g/L, pH 8 at T = 30 °C).

The initial concentration of dye in industrial wastewater is a critical parameter that significantly influences the removal efficiency. The effect of MB concentration was studied at the optimized contact time (40 min) and at equilibrium with conditions of pH 8, a temperature of 30 °C, and an agitation speed of 120 rpm, as shown in Fig. 12. The amount of nanocomposite was kept constant at 1.2 g/L, while the dye concentration was varied between 5 and 85 mg/L. It was observed that the equilibrium adsorption capacity (q_e_) increased significantly from 4.79 to 56.5 mg/g as the initial MB concentration rose from 5 to 85 mg/L. Conversely, the removal efficiency decreased from 99.72 to 24.27% within 40 min. At lower dye concertation, there are plenty of available active sites on the bead surface, allowing for efficient uptake of dye molecules via photo-induced adsorption. As the concentration increases, more dye molecules compete for the limited sites. At very high concentration, not all dye molecules can find any available sites. Furthermore, the dye molecules exert a screening effect, which limits the penetration of visible light through the solution, thereby reducing the photo-activation of the Ag_2_Mo_2_O_7_ phase. This leads to a decrease in removal efficiency to 24.22% at 85 mg/L within 40 min^6^. The maximum adsorption capacity (q_e_) was found to be 46.5 mg/g at an initial concentration of 85 mg/L. These results showed that the concentration of 13.86 mg/L was chosen as the optimal initial concentration of MB for future experimental evaluations. The removal percentage at the optimal concentration reached 91.78% within only 40 min, compared to 98% at its equilibrium. This rapid uptake highlights the superior kinetic performance of the SA/Al_2_O_3_/Ag_2_Mo_2_O_7_ microbeads.

#### Impact of pH

**Fig. 13:**
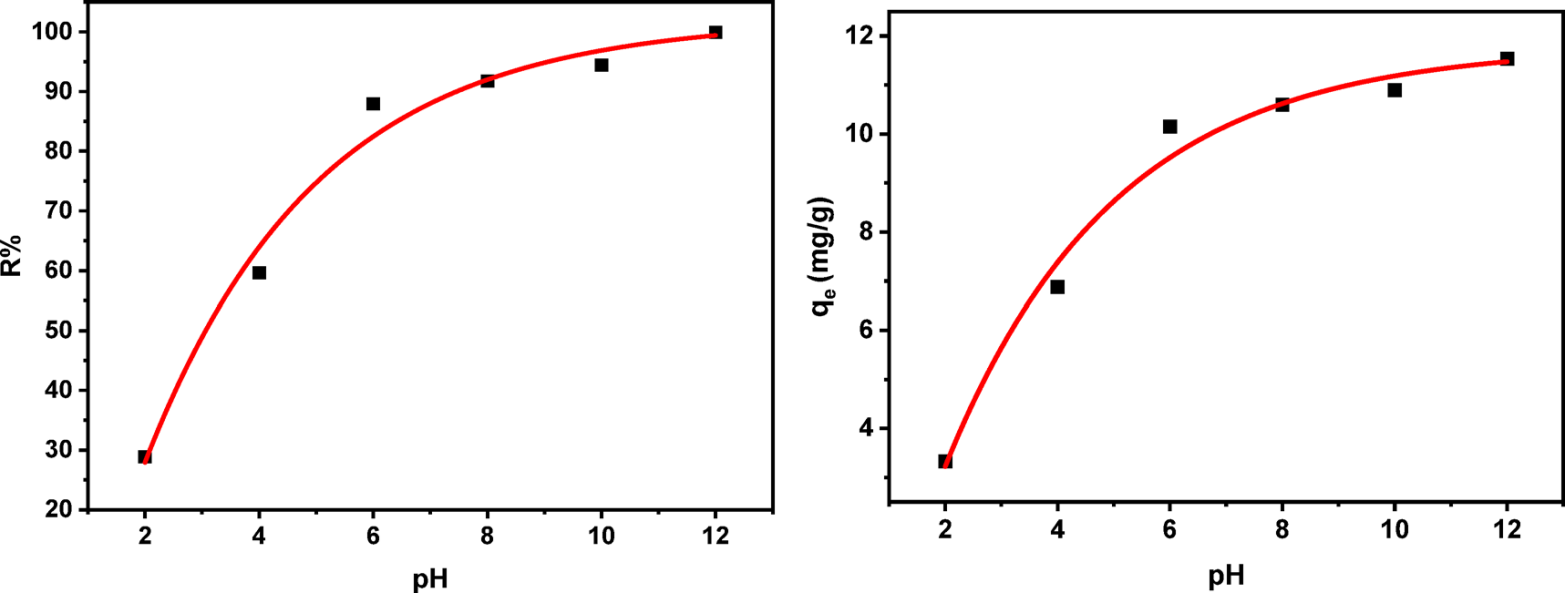
Effect of pH on the MB removal by SA/Al_2_O_3_/Ag_2_Mo_2_O_7_ nanocomposite microbeads. ([MB]_o_ = 13.86 mg/L, dose = 1.2 g/L at 30 °C).

The pH is a crucial parameter that governs the effectiveness of the photo-induced adsorption process by influencing the surface charges of the nanocomposite. Consequently, the removal of methylene blue (MB) dye by the ternary SA/Al_2_O_3_/Ag_2_Mo_2_O_7_ nanocomposite was investigated across a wide pH range of 2–12, keeping all other parameters fixed. The solution pH significantly alters the interactions between the dye molecules and the nanocomposite surface, affecting the photo-induced adsorption performance. It was evident that the removal efficiency diminished considerably with reducing pH, as illustrated in Fig. 13. The maximum photo efficiency was 99.94% at pH 12, which decreased to 28.87% at pH 2. The results indicate that the nanocomposite demonstrates an enhanced affinity for MB molecules in alkaline media^55^. This is primarily attributed to the direct photolysis and deprotonation of functional groups (such as carboxyl groups in the sodium alginate matrix) at higher pH values, which increases the negative charge density on the microbeads surface. This strengthens the electrostatic attraction between the negatively charged adsorbent and the cationic MB molecules. Conversely, at low pH, the abundance of H^+^ ions lead to surface protonation and competes with MB molecules for active sites, reducing the removal efficiency.

However, pH 8 was selected as the optimal value for all subsequent experimental investigations. This selection is based on a pragmatic balance between high removal efficiency (91.78%) and economic-environmental considerations. Operating at pH 8 is more sustainable for large-scale industrial applications as it is closer to the near-neutral pH of typical textile effluents. Thereby it minimizes the consumption of chemical reagents required for pH adjustment. This moderate pH enhances the stability of the SA/Al_2_O_3_/Ag_2_Mo_2_O_7_ microbeads for long-term reuse.

#### Impact of temperature

**Fig. 14:**
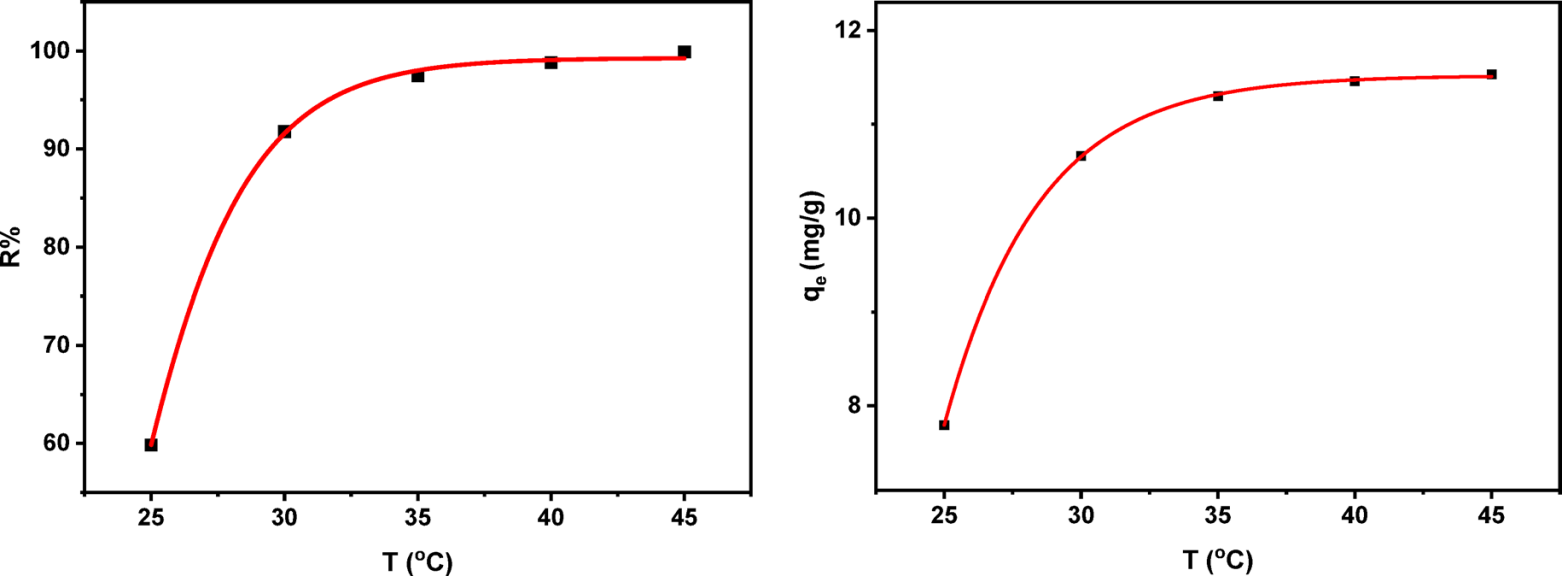
Effect of temperature on the MB removal efficiency (R%) and equilibrium adsorption capacity (q_e_)*.* (conditions: [MB]_o_ = 13.86 mg/L, dosage = 1.2 g/L, pH 8, and contact time = 40 min).

The influence of temperature change on the removal of MB was investigated over a temperature range of 25–45 °C, as shown in Fig. 14. It was observed that increasing the temperature significantly improved the removal efficiency, rising from 59.84% at 25 °C to nearly complete removal at 99.89% when the temperature reached 45 °C. Notably, the removal performance achieved high level at 45 °C within only 20 min, demonstrating exceptionally repaid kinetics at elevated temperatures. This enhancement can be attributed to the increased kinetic energy of the MB molecules at high temperatures, which facilitates their diffusion and interaction with the external surface and into the pores of the SA/Al_2_O_3_/Ag_2_Mo_2_O_7_ microbeads. This indicates that this photo-induced adsorption is endothermic process in nature. Although the highest performance occurred at 45 °C, the temperature 30 °C was maintained as the optimal temperature for all studies to balance high efficiency and energy saving consideration. This study provides the required data to calculate thermodynamic parameters.

### Kinetic studies and Thermodynamic parameters

**Fig. 15:**
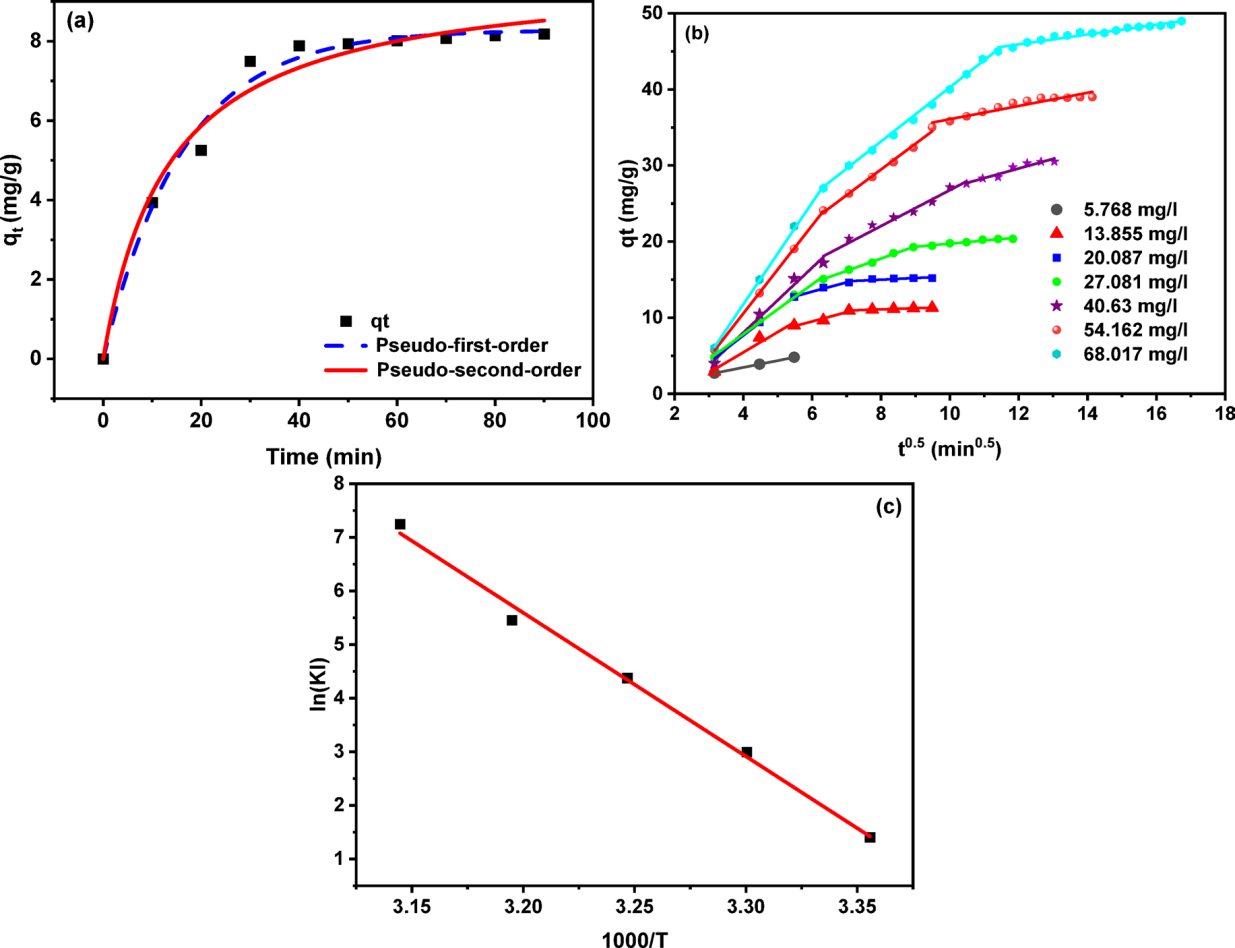
(**a**) Adsorption Kinetic models of MB on SA/Al_2_O_3_/Ag_2_Mo_2_O_7_ nanocomposite microbeads. [MB]_o_ = 13.86 mg/L, dose = 1.2 g/L at 30 °C. (**b**) Intraparticle diffusion model of MB on SA/Al_2_O_3_/Ag_2_Mo_2_O_7_ microbeads. Dose = 1.2 g/L at 30 °C. (**c**) Van’t Hoff equation.

The mechanism of MB photo-induced adsorption or removal process can be clarified through kinetic models. This helps identify the nature of the interaction between the adsorbent and adsorbate molecules, distinguishing whether the process is physical or chemical adsorption. Several kinetic models were applied including the Pseudo-first-order (PFO) and Pseudo-second-order (PSO). The PFO model assumes that the photo-induced adsorption occurs mainly through physisorption which involves weak forces such as Van der Waals interactions. In contrast, the PSO model suggests that the removal was chemisorption that chemical bonds between adsorbent and adsorbate^56^. Figure 15 presents the diagram of photo-induced adsorption kinetic of MB on the nanocomposite, while Table 3 summarizes the kinetic parameters. According to these results, the correlation coefficient (R^2^) value for PSO model, which were slightly higher than those for the PFO model. Furthermore, the calculated equilibrium capacity (q_e_) from the PSO model closely matched the experimentally measured value (q_e,exp_). These findings indicate that the photo-induced adsorption of MB onto the nanocomposite follows PSO model, suggesting different layers by chemisorption.

**Table 3:** Adsorption kinetics models for the removal of MB onto SA/Al_2_O_3_/Ag_2_Mo_2_O_7_.

Kinetic model	Initial concentration of MB (mg/L)						
	5.77	13.86	20.09	27.08	40.63	54.16	85.13
q_e, exp_ (mg/g)	4.79	11.3	15.2	20.36	30.5	38.9	56.5
Pseudo-first-order							
q_e,cal_ (mg/g)	5.5	11.2	15.9	20.6	31.5	40.3	45.4
k_1_ (min^−1^)	0.064	0.07	0.046	0.031	0.019	0.02	0.012
R^2^	0.997	0.999	0.970	0.990	0.992	0.997	0.930
Pseudo-second-order							
q_e,cal_ (mg/g)	7.83	12.99	20	25.7	34.1	40.5	58.2
k_2_ (g/mg min)	0.006	0.007	0.002	0.001	0.0008	0.0006	0.0004
R^2^	0.999	0.994	0.972	0.99	0.993	0.990	0.930
Intraparticle diffusion							
kp_1_ (mg/g min^1/2^)	0.902	2.67	3.516	3.278	4.273	5.789	6.689
R^2^	0.999	0.963	0.999	0.992	0.984	0.999	0.998
kp_2_ (mg/g min^1/2^)	–	1.213	1.218	1.619	2.362	3.378	3.579
R^2^	–	0.954	0.98	0.997	0.973	0.992	0.997
kp_3_ (mg/g min^1/2^)	–	0.151	0.21	0.407	1.263	0.867	0.645
R^2^	–	0.99	0.703	0.965	0.928	0.904	0.946

The mass transfer kinetics during the photo-induced adsorption were further explored using the intraparticle diffusion model. By plotting q_t_ against t^1/2^ as shown in Fig. 15, it became evident that the removal process doesn’t follow a simple single step. The initial sharp slope represents the rapid migration of MB from the bulk solution to the adsorbent surface. This is followed by a distinct stage, which the diffusion of the dye molecules into the pores of the nanocomposite. Finally, the third stage reflects the equilibrium phase, where the diffusion rate slows down as the available active sites within the nanocomposite become saturated^43^.

To further evaluate the photo-efficiency of the SA/Al_2_O_3_/Ag_2_MoO_7_ microbeads, the Apparent Quantum Yield (AQY) was calculated based on the MB removal data. For a 25 mL solution with an initial concentration of 13.86 mg/L, the AQY was found to be 0.41% after 40 min of irradiation (removal efficiency 92.35%) and 0.17% after 100 min (98.07% removal). The decrease in AQY over time is attributed to the reduction in available dye molecules and the gradual saturation of the surface-active sites. These values confirm the significant role of the visible light in enhancing the surface adsorption capacity of the ternary nanocomposites.

The removal mechanism can be analyzed through thermodynamic parameters such as the enthalpy change (ΔH°) and entropy change (ΔS°), which are typically derived from the Van’t Hoff equation.

$${\mathrm{Ln}}\left( {{\mathrm{k}}_{{\mathrm{L}}} } \right) = \frac{{{\Delta {\mathrm{S}}}^\circ }}{{\mathrm{R}}} - \frac{{{\Delta {\mathrm{H}}}^\circ }}{{{\mathrm{RT}}}}$$

Thermodynamic parameters are evaluated by calculating the Gibbs free energy change (ΔG°), which is expresses as

$$\Delta {\mathrm{G}}^{^\circ } = \Delta {\mathrm{H}}^{^\circ } - {\mathrm{T}}\Delta {\mathrm{S}}^{^\circ }$$

In the present study, the equilibrium constantly increased with rising temperature, indicating an endothermic adsorption process. The negative values of ΔG° confirm that the adsorption of MB onto nanocomposite is spontaneous and thermodynamically favorable. Additionally, the shift toward more negative ΔG° values with increasing temperature suggests enhanced removal efficiency and feasibility at higher temperatures. The range of ΔG° values can also provide further insight into the nature of photo-induced adsorption mechanism. Based on the presented results, the removal process aligns with strong chemical adsorption. The positive enthalpy change (ΔH°) supports the endothermic nature of the remediation process^57^ (Table 4).

**Table 4:** Thermodynamic parameters for MB removal onto SA/Al_2_O_3_/Ag_2_Mo_2_O_7_ nanocomposite.

Parameter	Temperature (K)				
	298	303	308	313	318
K_L_	4.07	19.98	79.45	233.33	1404.18
ΔG° (kJ/mol)	− 3.48	− 7.54	− 11.20	− 14.19	− 19.16
ΔH° (KJ/mol)	222.81				
ΔS° (J/mol K)	759.48				

### Isotherm models

**Fig. 16:**
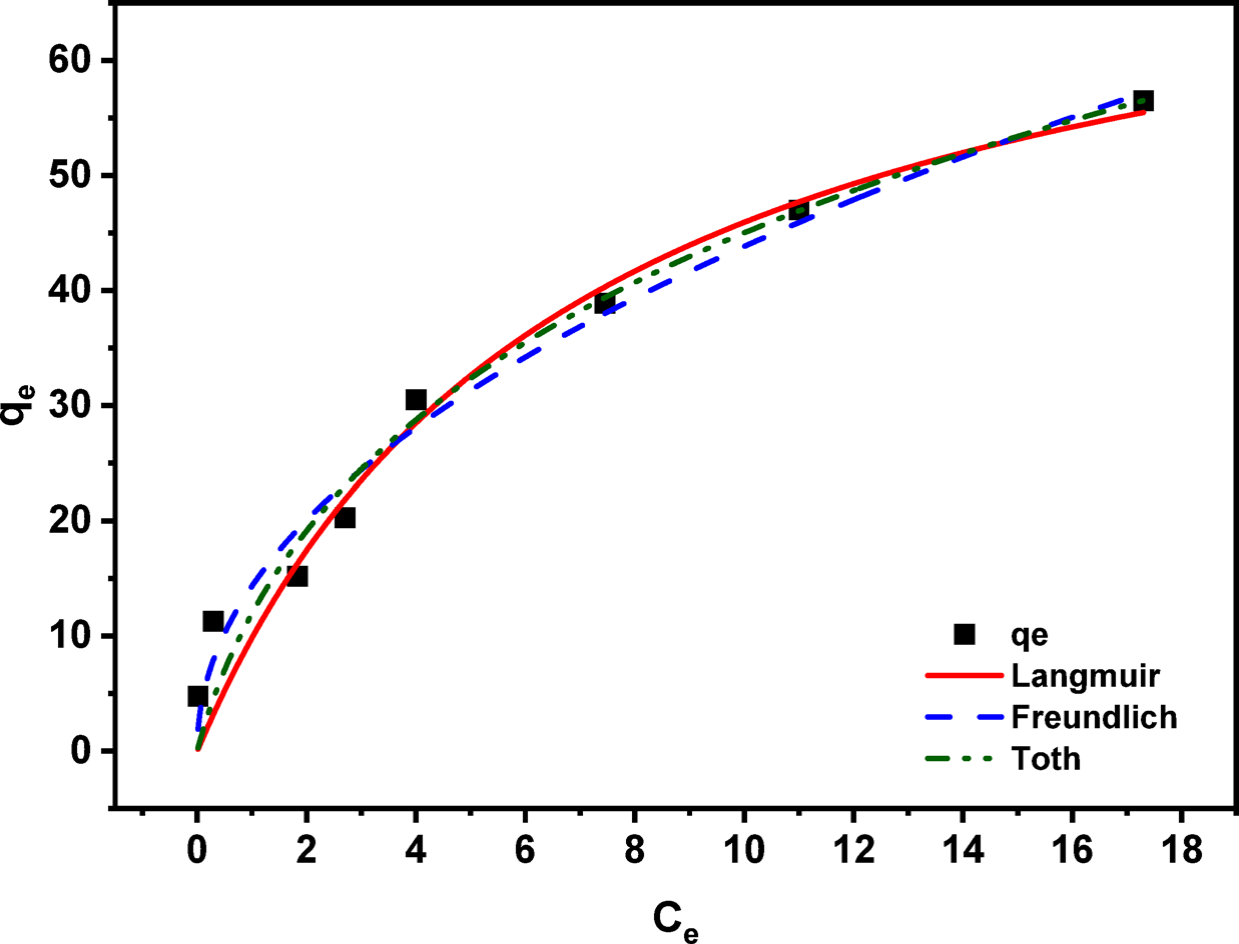
Non-linear plots of isotherm models of MB using SA/Al_2_O_3_/Ag_2_Mo_2_O_7_.

The removal behavior of MB onto the SA/Al_2_O_3_/Ag_2_Mo_2_O_7_ surface was evaluated through various isotherm models. These models decode the nature of the interactions between the nanocomposite and MB at a constant temperature. In this study, the Langmuir, Freundlich, and Toth models were employed to analyze the experimental data, as shown in Fig. 16. The calculated parameters and the corresponding correlation coefficient (R^2^) are summarized in Table 5. The Langmuir model assumes that the photo-induced adsorption occurs on a uniform surface, which each molecule forms a single layer^58^. The nonlinear form of this model is expressed as:

$${\mathrm{q}}_{{\mathrm{e}}} = \frac{{{\text{ q}}_{{\text{m }}} {\text{ K}}_{{\text{L }}} {\text{ C}}_{{\mathrm{e}}} }}{{1 + {\mathrm{k}}_{{\text{L }}} {\text{ C}}_{{\mathrm{e}}} }}$$

where q_m_ is the maximum capacity and K_L_ is the Langmuir constant. To check if the process is favorable, the separation factor (R_L_) was calculated. In our case, the R_L_ value was 0.332. Since this value is between 0 and 1, it confirms that the photo-induced adsorption of MB is favorable^59^. On the other hand, the Freundlich model describes photo-induced adsorption on irregular surfaces where multiple layers can form. It is expressed by the equation:

$${\mathrm{q}}_{{\mathrm{e}}} = {\mathrm{K}}_{{\text{F }}} {\text{ C}}_{{\mathrm{e}}}^{{1/{\mathrm{n}}_{{\mathrm{F}}} }} .$$

**Table 5:** Isotherm parameters for MB removal onto SA/Al_2_O_3_/Ag_2_Mo_2_O_7_ microbeads.

Isotherm model	Parameter	Value
Langmuir	q_m_ (mg/g)	77.5
	K_L_ (L/mg)	0.145
	R^2^	0.95
Freundlich	1/n_F_	0.485
	n_F_	2.06
	K_F_ (L/mg)	14.33
	R^2^	0.973
Toth	q_m_ (mg/g)	27.88
	K_t_	0.5
	T	0.73
	R^2^	0.999

The calculated 1/n_F_ value was 0.485, which also indicates a favorable process^60,61^. However, the R^2^ for Freundlich (0.973) was higher than (0.95) that of Langmuir. This suggests that the photo-induced adsorption of MB onto nanocomposite follows Freundlich model. This means that the removal process occurs on a heterogeneous surface with a non-uniform distribution of active sites.

The Toth isotherm is essentially an improved evolution of the Langmuir model. It developed to bridge the limitations in Langmuir model by incorporating the quasi-gaussian energy distribution theory. This approach provides more description of how molecules occupy a surface, specifically focusing on sub-monolayer coverage on active sites. The Toth equation is expressed as:

$${\mathrm{q}}_{{\mathrm{e}}} = \frac{{{\text{ q}}_{{\text{m }}} {\mathrm{K}}_{{\text{t }}} {\mathrm{C}}_{{\mathrm{e}}} }}{{\left[ {1 + ({\mathrm{k}}_{{\text{t }}} {\text{ C}}_{{\mathrm{e}}} )^{{\mathrm{t}}} } \right]^{{1/{\mathrm{t}}}} }}$$

The (t) parameter serves as critical diagnostic tool for evaluating surface homogeneity, where its value proximity to unity confirms that the energy distribution is remarkably uniform across the active sites. According to the results in Table 5, the Toth isotherm model exhibited the highest correlation coefficient (R^2^ = 0.999) compared to the Freundlich and Langmuir models. These suggests a complex adsorption behavior on a heterogenous surface. The Toth factor (t) was found 0.73, it confirms the presence of energetically non-equivalent sites on the surface with varied potential.

In summary, the Langmuir model suggested a high maximum adsorption capacity of 77.5 mg/g, which demonstrates the superior performance of the SA/Al_2_O_3_/Ag_2_Mo_2_O_7_ microbeads compared to many reported adsorbents. But the Toth model provided a more statistically reliable fit (R^2^ = 0.999). The Toth capacity of 27.88 mg/g is considered a more realistic representation of the sequestration process, accounting for the energetic heterogeneity of the ternary nanocomposite surface. This heterogeneity is consistent with the presence of multiple active phases within the microbead architecture. Since the Langmuir model remains the standard benchmark in most published literature, its theoretical maximum capacity (q_max_) of 77.5 mg/g is utilized for comparative analysis. This allows for a fair evaluation against reported evaluation.

### The expected mechanism

The removal performance of the cationic MB molecules by using SA/Al_2_O_3_/Ag_2_Mo_2_O_7_ hybrid system is attributed to a sophisticated photo-induced adsorption mechanism^62^, characterized by the visible light induced electronic modulation of the surface. While the SA and alumina provide a porous framework for the initial dye capture, the poly silver molybdate serves as a photo-active sensitizer. Upon visible-light irradiation, the semiconductor phase (Ag_2_Mo_2_O_7_) undergoes electronic excitation, promoting electrons from the valence band to the conduction band. These photo-generated electrons (e^−^) rapidly migrate to the microbeads’ surface, effectively increasing the negative surface charge density. Since methylene blue (MB) is a cationic dye, the negatively excited surface of the microbeads is strongly attracted the cationic MB molecules toward them through the electrostatic attraction. This leads to an electronic pulling effect that accelerates the sequestration of dye molecules into the internal pores. The persistent blue coloration of the beads post-treatment confirms that the process is a high-efficiency physical–chemical process enhanced by light. This hybrid system ensures rapid treatment with minimal energy and material demand.

The photo-induced adsorption of MB onto microbeads is driven by the following interactions. Firstly, electrostatic attractions such as the cationic methylene blue (MB^+^) molecules are strongly attracted to the abundant anionic sites on the surface, specifically the carboxylate (COO^−^) and hydroxyl (OH^−^) groups of the sodium alginate. Additionally, pore diffusion step, where the Al_2_O_3_ framework acts as a rigid and porous scaffold that facilitates the transport of dye molecules from the aqueous solution into the internal pores of the microbeads. Finally, the molecular interactions such that the photo-adsorption is further stabilized by hydrogen bonding between the nitrogen atoms of MB and the hydroxyl groups of the composite, as well as $\pi$–$\pi$ interactions between the aromatic rings of the MB dye and the functionalized surface of the composite.

In conclusion, light turns our material into a stronger electronic sponge that captures the dye molecules inside its pores and active sites faster, making it a highly efficient, light powered sponge for water cleaning.

### Reusability

**Fig. 17:**
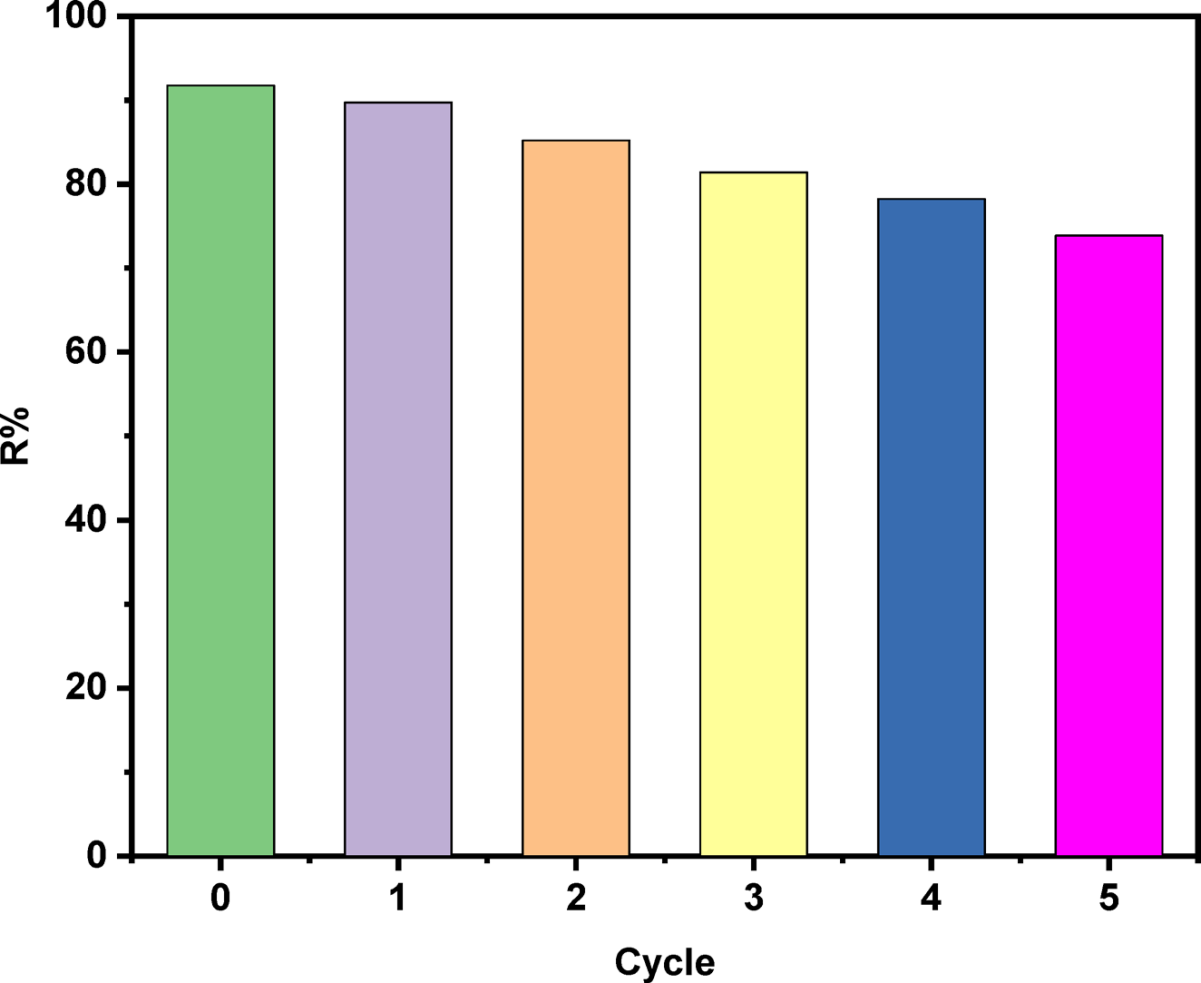
Reusability of SA/Al_2_O_3_/Ag_2_Mo_2_O_7_ microbeads for MB removal. [MB]_o_ = 13.86 mg/L, Dose = 1.2 g/L at 30 °C.

In many applications, discarding the adsorbent as waste is not financially reasonable. Reuse tests were conducted under the same removal conditions. A key challenge for newly designed composite to be practically viable, they must exhibit high stability and consistent reusability through series cycles^63^. The results indicated that the beads maintained satisfactory removal efficiency even after five repeated cycles, as shown in Fig. 17. However, the removal declined from 91.78 to 73.87% over these cycles. To assess environmental safety, metals analysis was conducted to monitor ion leaching (Al, Ag, Mo) after the removal process. The concertation of leaked ions remained below 0.5 mg/L (0.01 mg/L for Ag, 0.19 mg/L for Al and 0.45 mg/L for Mo), confirming the high structural integrity and stability of the SA/Al_2_O_3_/Ag_2_Mo_2_O_7_ microbeads. The sustained performance of the SA/Al_2_O_3_/Ag_2_Mo_2_O_7_ microbeads over multiple cycles highlights their primary advantages as a promising photo-responsive adsorbent. The nanocomposite demonstrates high stability behavior, indicating its potential as an efficient and reusable nanocomposite for industrial wastewater treatment applications.

### Comparative study

This study presents a comparative analysis of the synthesized ternary nanocomposite against other established composites for the removal of methylene blue (MB) from aqueous solutions. This evaluation highlights a strategic balance between removal efficiency and industrial feasibility. Specifically, the SA/Al_2_O_3_/Ag_2_Mo_2_O_7_ nanocomposite microbeads offer significant advantages in terms of mechanical stability, rapid photo-induced adsorption synergy, and good reusability. Unlike traditional nano-powders, the macroscale design of these beads prevents secondary nanoparticle pollution and ensures easy mechanical recovery. While the macroscale introduces minor mass transfer resistance compared to fine powders, the high photo-induced adsorption synergy effectively compensates for these limitations. Furthermore, there are some drawbacks such as lower surface area compared to fine powders, cost of silver precursors, limited mineralization, and limited deep light penetration into the core of microbead. The use of visible light as a sustainable energy source and the green synthesis approach enhances the economic and environmental viability of the system for large-scale applications.

To evaluate the performance of the fabricated ternary nanocomposite, a comparative study was conducted against various recently reported adsorbents for MB removal, as summarized in Table 6. The data demonstrates that our SA/Al_2_O_3_/Ag_2_Mo_2_O_7_ photo-responsive adsorbent exhibit a highly removal efficiency, achieving 91.78% at pH 8 and reaching 99.94% at pH 12 under visible light in 40 min. However, in the absence of light (dark adsorption), the nanocomposite act as a conventional adsorbent, achieving a removal efficiency of 81.6% in 40 min. These results clearly demonstrate that our novel nanocomposite exhibits a superior efficiency for MB removal within a shorter treatment time compared to other reported adsorbents. Overall, the comparative study confirms the promising application of the SA/Al_2_O_3_/Ag_2_Mo_2_O_7_ nanocomposite in wastewater treatment^64–71^.

**Table 6:** Comparison of maximum adsorption capacities of SA/Al_2_O_3_/Ag_2_Mo_2_O_7_ photo-responsive adsorbent and other adsorbents for the removal of MB.

Adsorbent/photo-adsorbent	Time (min)	R%	q_max_ (mg/g)	Refs.
SA/Al_2_O_3_/Ag_2_Mo_2_O_7_ photo-responsive adsorbent	40	91.78	77.5	Current study
TiO_2_/CQDs/Alg	90	68	44.13	^64^
Bentonite/SDBS	10	–	23.54	^65^
Pristine chitosan	30	80	7.61	^66^
Biochar	360	–	7.2	^67^
Fe_3_O_4_/biochar photo-adsorbent	30	88	0.02	^68^
Chitosan/laterite/iron oxide	1440 or 24 h	93.7	11.5	^69^
Graphene oxide/citric acid/sodium alginate	120	87.5	175	^70^
PANI/cadmium ferrite	40	85	17.92	^71^

## Conclusion

The present study successfully addresses the urgent requirement for sustainable water remediation technologies by fabricating an eco-friendly SA/Al_2_O_3_/Ag_2_Mo_2_O_7_ photo-responsive nanocomposite via a facile green inotropic gelation route. Structural characterization confirmed its heterogeneous nature with excellent stability and good surface area. The experimental data revealed that the ternary nanocomposite serves as a high-performance photo-responsive adsorbent, capable of removing 91.78% of MB under optimized conditions of 30 °C, pH 8, 40 min, and adsorbent dosage of 1.2 g/L. Crucially, the photo-efficiency was quantitatively validated by the Apparent Quantum Yield (AQY), which was determined to be 0.41% at 40 min. The kinetic studies indicated that the removal process followed pseudo-second-order model with a high correlation coefficient (R^2^ = 0.999). Furthermore, the recyclability experiments demonstrated excellent mechanical stability and reusability of microbeads even after 5 cycles, supported by negligible ion leaching of Al, Ag, and Mo. The ion leaching data confirms the material’s environmental safety. Finally, the present study focuses on the photo-induced adsorption of MB dye, future research will explore the stability of SA/Al_2_O_3_/Ag_2_Mo_2_O_7_ microbeads in continues flow systems or real wastewater and their performance against diverse industrial effluents. This will provide a broader understanding of the material’s scalability for large-scale wastewater treatment applications. In summary, the synthesized SA/Al_2_O_3_/Ag_2_Mo_2_O_7_ microbeads offer a sustainable, green, eco-friendly, and efficient solution for industrial wastewater treatment, characterized by high reusability and easy separation.

## Data Availability

All data generated or analyzed during this study are included in this published article.
